# *Caenorhabditis elegans* PTR/PTCHD PTR-18 promotes the clearance of extracellular hedgehog-related protein via endocytosis

**DOI:** 10.1371/journal.pgen.1009457

**Published:** 2021-04-19

**Authors:** Hirohisa Chiyoda, Masahiko Kume, Carla Cadena del Castillo, Kenji Kontani, Anne Spang, Toshiaki Katada, Masamitsu Fukuyama

**Affiliations:** 1 Department of Physiological Chemistry, Graduate School of Pharmaceutical Sciences, The University of Tokyo, Tokyo, Japan; 2 Biozentrum, University of Basel, Basel, Switzerland; Rutgers The State University of New Jersey, UNITED STATES

## Abstract

Spatiotemporal restriction of signaling plays a critical role in animal development and tissue homeostasis. All stem and progenitor cells in newly hatched *C*. *elegans* larvae are quiescent and capable of suspending their development until sufficient food is supplied. Here, we show that *ptr-18*, which encodes the evolutionarily conserved patched-related (PTR)/patched domain-containing (PTCHD) protein, temporally restricts the availability of extracellular hedgehog-related protein to establish the capacity of progenitor cells to maintain quiescence. We found that neural progenitor cells exit from quiescence in *ptr-18* mutant larvae even when hatched under starved conditions. This unwanted reactivation depended on the activity of a specific set of hedgehog-related *grl* genes including *grl-7*. Unexpectedly, neither PTR-18 nor GRL-7 were expressed in newly hatched wild-type larvae. Instead, at the late embryonic stage, both PTR-18 and GRL-7 proteins were first localized around the apical membrane of hypodermal and neural progenitor cells and subsequently targeted for lysosomal degradation before hatching. Loss of *ptr-18* caused a significant delay in GRL-7 clearance, causing this protein to be retained in the extracellular space in newly hatched *ptr-18* mutant larvae. Furthermore, the putative transporter activity of PTR-18 was shown to be required for the appropriate function of the protein. These findings not only uncover a previously undescribed role of PTR/PTCHD in the clearance of extracellular hedgehog-related proteins via endocytosis-mediated degradation but also illustrate that failure to temporally restrict intercellular signaling during embryogenesis can subsequently compromise post-embryonic progenitor cell function.

## Author summary

Inside the animal body, many “signals” are exchanged between tissues and cells to ensure healthy growth and physiological conditions. Too much or less signals have been known to cause various diseases such as cancer and diabetes. When developing animals are not fed enough food, their growth rates generally slow down. We found that when nematodes lacking the PTR-18 protein are hatched out of the egg in the absence of sufficient food, they do not appropriately halt their growth. Analyses at the molecular and cellular levels showed that immediately before hatching, PTR-18 re-uptakes a signaling molecule called GRL-7, which is known to promote growth, from the outside to the inside of cells, resulting in the degradation of GRL-7. As a result, worms lacking PTR-18 still contain excess GRL-7 after hatching, which results in unwanted growth even when ample food is not available. Thus, our findings uncover the role of PTR-18 in terminating a signal on time. Humans also possess a protein called PTCHD1, which is structurally similar to the worm’s PTR-18 and has been proposed to cause autistic spectrum disorders and learning disabilities. Our findings may provide a clue to further understand the function of human PTCHD1.

## Introduction

*C*. *elegans* L1 larvae hatch out of their eggshells with quiescent stem and progenitor cells. Sufficient food supply initiates L1 development by coordinating the release of stem and progenitor cells from quiescence [1–4]. Conversely, under nutritionally unfavorable conditions, the newly hatched larvae enter developmental dormancy, called L1 arrest or L1 diapause, and survive for weeks until ample food becomes available [5]. Because of the ease of manipulating and tracking their quiescence and reactivation, stem and progenitor cells in *C*. *elegans* L1 larvae have served as excellent models to study the nutritional regulation of stem cells *in vivo*.

Previous studies have shown that the insulin/insulin-like growth factor signaling (IIS) pathway plays a critical role in the developmental decision in response to nutrient availability. For example, loss of *daf-16/foxo*, which results in the constitutive activation of the IIS pathway, causes unwanted reactivation of many types of somatic progenitor cells, such as the P neuronal and M mesodermal progenitor cells [2,6]. MicroRNA (miR)-235 acts partly downstream of the IIS pathway to regulate a subset of L1 developmental events during L1 arrest [7]. Expression analysis and rescue experiments suggested that the miRNA primarily acts in the hypodermis to suppress the reactivation of P and M progenitor cells [7]. These findings led us to identify *grl-5* and *grl-7* as target genes of miR-235 that mediate the non-autonomous regulation of P cell quiescence by the hypodermis [8]. Both *grl-5* and *grl-7* encode putative secreted proteins and belong to the *hedgehog*-related (*hh*-r) gene family, which has been proposed to have evolved from the same ancestral gene as hedgehog (Hh) in other animals [9]. Inhibition of *grl-5* and *grl-7* can partially suppress the inappropriate reactivation of P cells in starved *mir-235* larvae, suggesting that these *grl* genes promote cellular events.

Several genes in the Hh signaling pathway, such as *Hh*, *Smoothened*, *Cos2*, *Fused*, and *Suppressor of Fused* are absent in the *C*. *elegans* genome [10]. However, the nematode possesses two patched orthologs, *ptc-1* and *ptc-3* [11]. Knockdown of *ptc-3* and multiple *hh*-r genes results in molting defects, raising the possibility that these genes act together in the same genetic pathway [12,13]. In addition to *hh*-r and *ptc* genes, some of the *ptr* (*patched-related*) genes were also shown to result in similar molting defects [12,14]. PTR, also called patched domain-containing (PTCHD) proteins, are found in other species such as *Drosophila*, mouse, and human [11,15]. PTR/PTCHD contains a region that is conserved among the Niemann-Pick Type C proteins, which are involved in cholesterol transport, the Dispatched protein, which promotes the secretion of Hh protein, and the Hh receptor Patched [16–19]. This membrane-spanning region contains the “sterol-sensing domain,” which is involved in binding to and sensing cholesterol [19,20]. In addition, PTR/PTCHD, NPC1, Dispatched, and Patched proteins belong to the resistance, nodulation, and division (RND) transporter superfamily [21]. Members of the superfamily generally contain a 12-transmembrane (TM) domain, which is thought to have arisen from an intragenic duplication of a six-transmembrane domain [22], and a conserved GXXXD motif, which has been shown to play a critical role in the bacterial RND transporter activity [23,24]. Furthermore, Patched, Dispatched, and PTR/PTCHD proteins contain not only an expanded GXXXDD motif within TM4 but also a GXXXD/E motif within TM10 [13]. Introduction of mutations in Patched GXXXDD motif and in Dispatched GXXXDD and GXXXD/E motifs impairs their activities [13,25–27].

Although the *ptr/ptchd* genes are conserved among several animal species, little is known about their cellular functions. Mutations in the human *ptchd1* gene are found in patients with autistic spectrum disorders and learning disabilities [28–34]. Furthermore, *ptchd1* knockout mice show attention-deficit hyperactivity disorder (ADHD)-like phenotypes [35]. Loss of the *Drosophila ptr* gene results in embryonic lethality [36]. In addition to their involvement in molting, the functions of few *C*. *elegans ptr* genes have been reported in detail. For instance, *daf-6* is involved in the formation of the glial channel that surrounds the receptive endings of the sensory neurons and likely regulates vesicular transport [37–40]. *ptr-24* has been proposed to act downstream of the *hh*-r gene, *grl-21*, to regulate mitochondrial fragmentation and lipid accumulation [41]. Additionally, *wrt-10*, which belongs to another subfamily of *hh*-r genes, reportedly promotes oocyte quality maintenance and delays reproductive decline via *ptc-1* and *ptr-2* [42].

Here, we show that *C*. *elegans* PTR-18 promotes the clearance of extracellular Hh-related proteins via endocytosis-mediated degradation, potentially acting as its decoy receptor. Under nutrient-deficient conditions that force the wild-type larvae to enter L1 arrest, newly hatched *ptr-18* mutant L1 larvae show reactivation of P progenitor cells. This arrest-defective phenotype is suppressed by the inhibition of a particular set of *grl* genes, including *grl-5*, *grl-7*, and *grl-27*. Unexpectedly, analysis using reporter genes showed that neither PTR-18 nor GRL-7 were expressed in newly hatched larvae. Instead, these proteins are temporally localized along the periphery of the apical membranes of hypodermal and P neuronal progenitor cells during late embryogenesis and are subsequently targeted to lysosomal degradation before hatching. This temporally controlled clearance of GRL-7 requires activity of PTR-18, so that newly hatched *ptr-18* mutant larvae still exhibit extracellular GRL-7 accumulation. Furthermore, the GXXXDD motif within TM4, the GXXXD/E motif within TM10, and the cytoplasmic C-terminal portion of PTR-18 protein are indispensable for its appropriate function. These findings reveal a previously undescribed function of PTR/PTCHD as a sink for extracellular Hh-related proteins and illuminate the importance of the temporal regulation of extracellular signaling in maintaining progenitor cell function.

## Results

### *ptr-18* is required to maintain the quiescence of progenitor cells during L1 arrest

**Fig 1:**
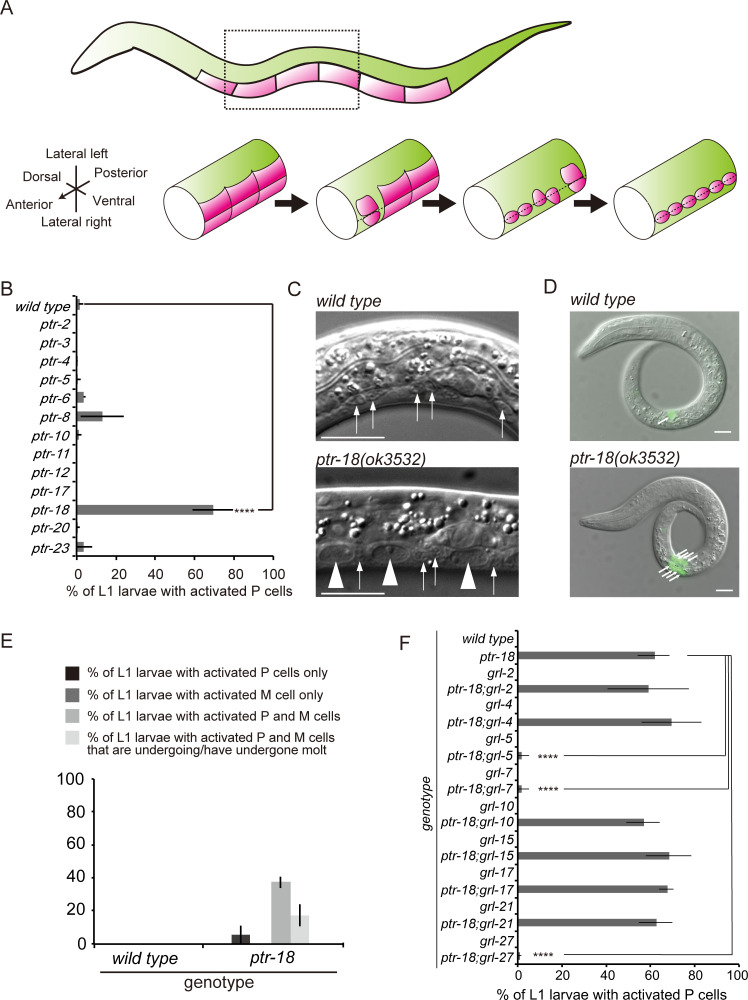
*ptr-18* is required for maintaining the quiescence of progenitor cells during L1 arrest. (A) Schematic of the reactivation of P neural progenitor cells. Drawings were made based on https://www.wormatlas.org/hermaphrodite/hypodermis/Hypframeset.html. Top: A newly hatched L1 larvae with quiescent P cells (indicated in magenta). Anterior is to the left, and dorsal is up. Bottom: A part of the body of developing L1 larvae. P cell reactivation is detected by their ventral migration. (B) Percentage of starved L1 larvae showing ≥1 reactivated P cell(s). P cells that had migrated to the ventral nerve cord after 5 days of L1 starvation were scored as “reactivated.” Experiments were repeated ≥4 times, and n≥35 animals were scored for each experiment. Data are presented as mean ± SD. ****: p <0.0001 (Fisher’s exact test). (C) Differential interference contrast photographs of wild-type and *ptr-18(ok3532)* L1 larvae after 5 days of L1 starvation. Only the cell bodies of motor neurons (arrows) were observed along the ventral nerve cord in the wild-type animals. In contrast, migrated P cells (arrowheads) were found along with the cell bodies in the *ptr-18* mutant animal. Scale bar: 10 μm. (D and E) Reactivation of M cells and molting occur in starved *ptr-18(ok3532)* L1 larvae. M cell and its descendants (arrows in Fig 1D) were visualized by P*hlh-8*::*gfp* [102]. Note that *ptr-18* mutant animals used for Fig 1B and 1C did not carry the transgene. Experiments were repeated ≥3 times, and n ≥50 animals were scored for each experiment. Data are presented as mean ± SD. Scale bar: 10 μm. (F) Genetic interactions between *ptr-18* and *grl* mutations. The indicated phenotype was scored after 5 days of L1 starvation. Data are presented as mean ± SD. Experiments were repeated three times, and n ≥35 animals were scored for each experiment. ****: p <0.0001 (Fisher’s exact test).

As shown in Fig 1A, six pairs of P neural progenitor cells first reside along the ventrolateral sides in newly hatched larvae. When the larvae are supplied with ample food, the most anterior pair of quiescent P cells migrate into the ventral nerve cord during the mid-L1 stage, followed successively by the more posterior pairs. This reactivation of quiescent P cells is easily detected under a differential interference contrast microscope [43]. Our previous studies showed that forced expression of the *hh*-r gene, *grl-7*, in starved L1 larvae can reactivate P neuroblasts [8]. In addition, *grl-7* and another *hh*-r gene, *grl-5*, partially mediate reactivation of P neuroblasts in starved *mir-235* mutant L1 larvae [8]. RNAi targeting some of the *hh*-r and *ptr* genes results in similar developmental defects, such as failure to complete molting and small body size [12–14], suggesting that these genes act in the same genetic pathway. Similarly, previous studies have proposed that *grl-21* negatively regulates *ptr-23* to regulate mitochondrial fragmentation and lipid accumulation [41]. Thus, we hypothesized that the *ptr* gene may be involved in maintaining the quiescence of P cells during L1 arrest by antagonizing the activity of *grl-5* and *grl-7*. We found that among the available *ptr* mutants, the majority of *ptr-18* mutant larvae showed reactivation of P cells when starved after hatching (Fig 1B and 1C). Further, we conducted RNAi against *ptr-2*, *4*, *6*, *9*, *11*, *13*, *14*, *16*, *17*, *19*, *20*, *22*, *23*, and *24*, and none of the starved L1 larvae from RNAi-treated mothers showed abnormal P cell reactivation (50 animals were scored; n = 1). Since *ptr-18* mutant animals exhibit a P cell defect at relatively high penetrance, we decided to focus our analysis on the *ptr-18* gene. In addition, reactivation of the mesoblast M cell and molting were observed in starved *ptr-18* mutant larvae (Fig 1D). As observed in *mir-235* mutant animals [7], animals that had undergone M cell proliferation always show migrated P cell(s), whereas molted animals always harbor reactivated P and M cells (Fig 1E), suggesting that P cells, M cell, and molt become activated in this strict order in starved *ptr-18* animals. In contrast, the primordial germ cells, Z2 and Z3, remain quiescent in *ptr-18* mutant larvae after 5-day L1 starvation (S1A Fig), similar to the *daf-16/foxo* and *mir-235* mutant animals [3,7]. When starved in cholesterol- and ethanol-free complete S medium after hatching, most of the *daf-16/foxo*-null mutant animals could not survive beyond 10 days (S1B Fig). In contrast, *ptr-18* mutant animals were relatively resistant to starvation stress, similar to the wild-type animals (S1B Fig).

### Activities of *grl-5*, *grl-7*, and *grl-27* are required for P cell reactivation in starved *ptr-18* mutant animals

Given the similarity of GRLs to Hh and PTR-18 to PTC, we examined whether GRL-5 and GRL-7 would act through PTR-18 or independently. To this end, we tested whether the activities of *grl-5* and *grl-7* contribute to the inappropriate reactivation of P cells in the starved *ptr-18* mutant larvae. Deletion mutations of these *grl* genes were introduced in *ptr-18* mutant animals. Unexpectedly, we found that the inhibition of both *grl-5* and *grl-7* almost completely suppressed the phenotype in the *ptr-18* mutant larvae, suggesting that *ptr-18* acts upstream of, but not downstream of, these *grl* genes (Fig 1F). Previous studies have shown that the inhibition of *grl-5* and *grl-7* activities only partially suppresses the defect in *mir-235* mutant larvae [8]. Thus, these observations suggest that the reactivation of P cells in *ptr-18* mutant animals is heavily dependent on the activity of these *grl* genes. Previous studies using reporter genes suggested that in addition to *grl-5* and *grl-7*, several other *grl* genes are expressed in the P and hypodermal cells [44]. Strikingly, the inhibition of *grl-27* comparably suppressed the phenotype in *ptr-18* mutant animals, similarly to *grl-5* and *grl-7* (Fig 1F). In contrast, the elimination of *grl-2*, *grl-4*, *grl-5*, *grl-10*, *grl-15*, *grl-17*, and *grl-21* activities did not significantly affect the defect (Fig 1F). These findings suggest that *ptr-18* antagonizes the activity of a specific subset of *grl* genes among those expressed in the P and hypodermal cells. Because *grl-5*, *grl-7*, and *grl-27* are required for P cell activation in starved *ptr-18* mutant animals, these *grl* genes might also play a critical role in the exit of P cells from quiescence in well-fed wild type animals. However, the triple mutant animals of *grl-5*, *grl-7*, and *grl-27* did not exhibit a delay in the timing of P cell activation under the fed condition (S1C Fig). These findings suggest that these *grl* genes do not contribute to P cell reactivation under the fed condition. Alternatively, an additional *hh*-r gene may act together with these *grl* genes.

### Spatiotemporal dynamics of PTR-18::GFP reporter expression

**Fig 2:**
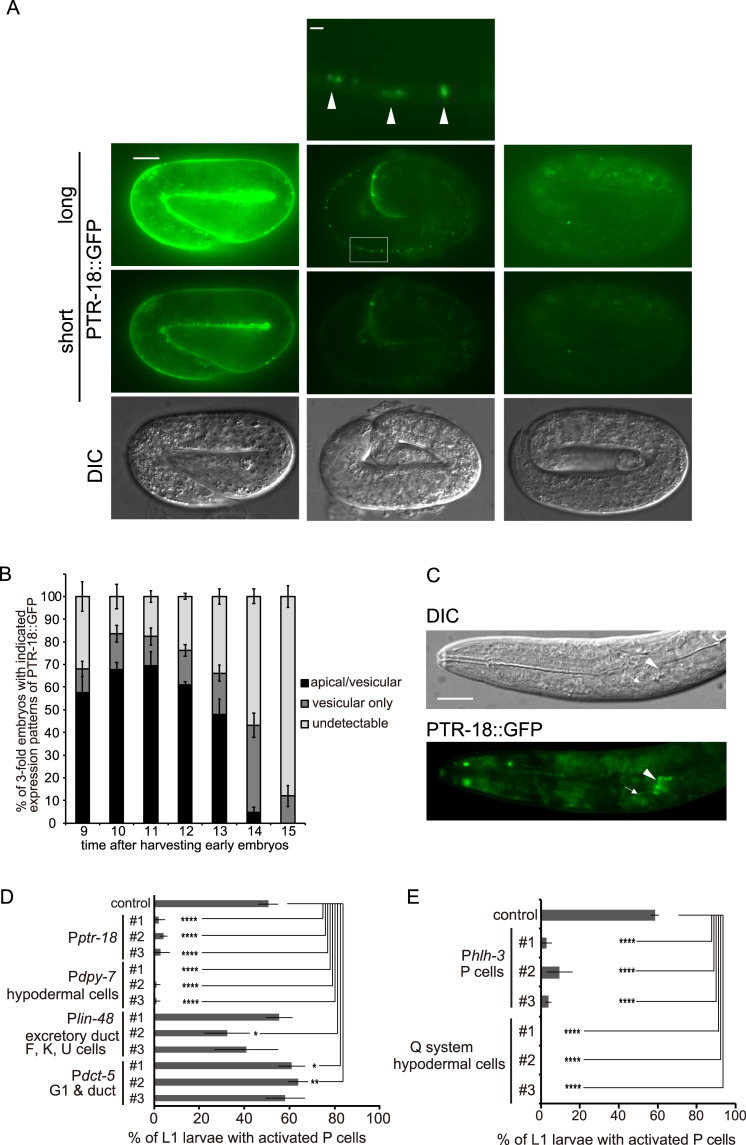
*ptr-18* acts in P and hypodermal cells. (A) Expression pattern of PTR-18::GFP during the 3-fold stage. Photographs were taken with fixed long and short exposure times, as indicated by “long” and “short.” Note that apical PTR-18::GFP distribution can be visualized by the short exposure, whereas vesicular PTR-18::GFP patterns become only conspicuous by the long exposure. PTR-18::GFP localized in either the apical side of surface cells that cover the whole body, which consists of hypodermal and P cells (left images; see also S4 Fig), or intracellular vesicular structures (middle images). The top panel is a magnified view of the image below (indicated by the rectangle), and the apical border of cells is indicated by arrowheads. A fraction of the 3-fold embryos carrying the transgene, marked by mCherry expression driven by the *ptr-18* promoter, did not show detectable expression of PTR-18::GFP (right images). Scale Bar: 10 μm (whole-embryo image) and 1 μm (magnified image). (B) Percentage of animals showing the indicated expression patterns were scored at the indicated times after harvesting early embryos. Data are presented as mean ± SD. Experiments were repeated three times, and ≥35 animals were scored for each time point. (C) Faint expression of PTR-18::GFP was detected in the excretory duct (arrowhead) and G1 pore (arrow) cells in L1 larvae after 24 h L1 starvation. Scale Bar: 10 μm. (D) Effects of PTR-18::VENUS expression on inappropriate reactivation of P cells in starved *ptr-18(ok3532)* mutant larvae. PTR-18::VENUS was expressed under the control of the promoters indicated (see S2D Fig). Animals that had lost the array expressing PTR-18::VENUS under the control of the *lin-48* promoter (line #1) were used as control. (E) Expression of PTR-18::VENUS in P or hypodermal cells restored the defect in maintaining P cell quiescence. PTR-18::VENUS was expressed in P or hypodermal cells using the *hlh-3* promoter or Q system, respectively (S2E–S2G Fig). YB3808 strain expressing *mcherry* under the control of the *dpy-7* promoter was used as control. For D and E, data are presented as mean ± SD. Experiments were repeated three times, and ≥35 animals were scored for each experiment. *: P<0.05, **: P <0.01, and ****: p <0.0001 (Fisher’s exact test).

To elucidate the expression pattern of *ptr-18*, we constructed a GFP translational reporter by inserting the *gfp* gene into the open reading frame of the *ptr-18* gene in the fosmid WRM0613dH03.1. This fosmid *ptr-18* reporter gene was introduced as an extrachromosomal array. Expression of PTR-18::GFP was bright enough for live imaging, though it should be noted that genes in the extrachromosomal array generally tend to be overexpressed. PTR-18::GFP was first detected along the apical side of surface cells that cover the whole body, which consists of hypodermal, seam, and P cells at the 3-fold stage during embryogenesis (Fig 2A; also see S4 Fig below). To determine the localization pattern of PTR-18::GFP in 3-fold embryos in detail, we dissolved the gravid, transgenic animals to harvest early embryos and allowed them to grow synchronously. Transition of the population ratio of 2-fold embryos, 3-fold embryos, and L1 larvae from 9 to 16 h after harvest confirmed the developmental synchronization of the collected embryos (S2A Fig). During the early time point, the majority of the 3-fold embryos initially showed apical localization of PTR-18::GFP (Fig 2A and 2B). As development continued, the percentage of the apical localization decreased, the fraction that exhibited localization of PTR-18::GFP in the vesicular punctate structures increased, and eventually, most of the embryos did not show its expression (Fig 2A and 2B). These observations indicate that PTR-18::GFP first localizes at the apical side of the hypodermal, seam, and P cells, subsequently accumulates in the vesicular structures, and eventually disappears before hatching. Unexpectedly, despite the robust phenotypes of *ptr-18(ok3532)* animals during L1 arrest, faint expression of PTR-18::GFP was only occasionally observed in the excretory duct and G1 pore cells during L1 starvation (Fig 2C). Expression of PTR-18::GFP only reappeared along the apical surface of the hypodermal, seam and P cells 11 h after the L1-arrested larvae were fed, which was several hours after P cell reactivation was initiated (S2B Fig). These observations raise the possibility that *ptr-18* acts before hatching to regulate the quiescence of P cells. PTR-18::GFP was also expressed in the descendants of P cells (S2B Fig; arrows), rectal epithelial F, K, and U cells (S2B Fig; arrowhead), and some seam cells in late L1 larvae (S2C Fig; red arrowheads).

### *ptr-18* acts in P and hypodermal cells

There is an open reading frame, Y38F1A.4, within the third intron of the *ptr-18* gene. However, the expression of *ptr-18 cDNA*::venus fusion gene under the control of its native promoter almost completely suppressed the inappropriate reactivation of P cells in starved *ptr-18* mutant larvae, indicating that the observed defects were caused by the reduced activity of *ptr-18* and not of Y38F1A.4 (Fig 2D).

To determine the site of *ptr-18* action, *ptr-18 cDNA*::*venus* was expressed under the control of promoters whose activities were specific to the cells and tissues where the *ptr-18*::*gfp* was expressed (S2D Fig). When *ptr-18 cDNA*::*venus* was expressed under the control of the *dpy-7* promoter, which is active in hypodermal, part of seam, and P cells [45], it rescues the P cell activation defect of *ptr-18* mutant animals as efficiently as *ptr-18 cDNA*::*venus* driven by the native promoter (Fig 2D). In contrast, the phenotype was hardly affected when *ptr-18*::*venus* was expressed under the control of the *lin-48* promoter, which is active in the excretory duct as well as F, K, U cells [46], and the *dct-5* promoter, which was previously used to mark the G1 and duct cells [47]. Furthermore, the expression of *ptr-18*::*venus* in either P or hypodermal and some seam cells driven by the *hlh-3* promoter [48] and Q system [49], respectively, could efficiently suppress the phenotype (Figs 2E and S2E–S2G), suggesting that *ptr-18* acts both autonomously and non-autonomously to maintain the quiescence of P cells. Because PTR-18::GFP was detected in the hypodermal, seam, and P cells only during the 3-fold stage before P cells become reactivated (Fig 2A and 2B; see also S4 Fig below), *ptr-18* acts before hatching to regulate the quiescence of P cells.

### GRL-5 and GRL-7 reporter proteins show expression patterns similar to that of PTR-18::GFP

**Fig 3:**
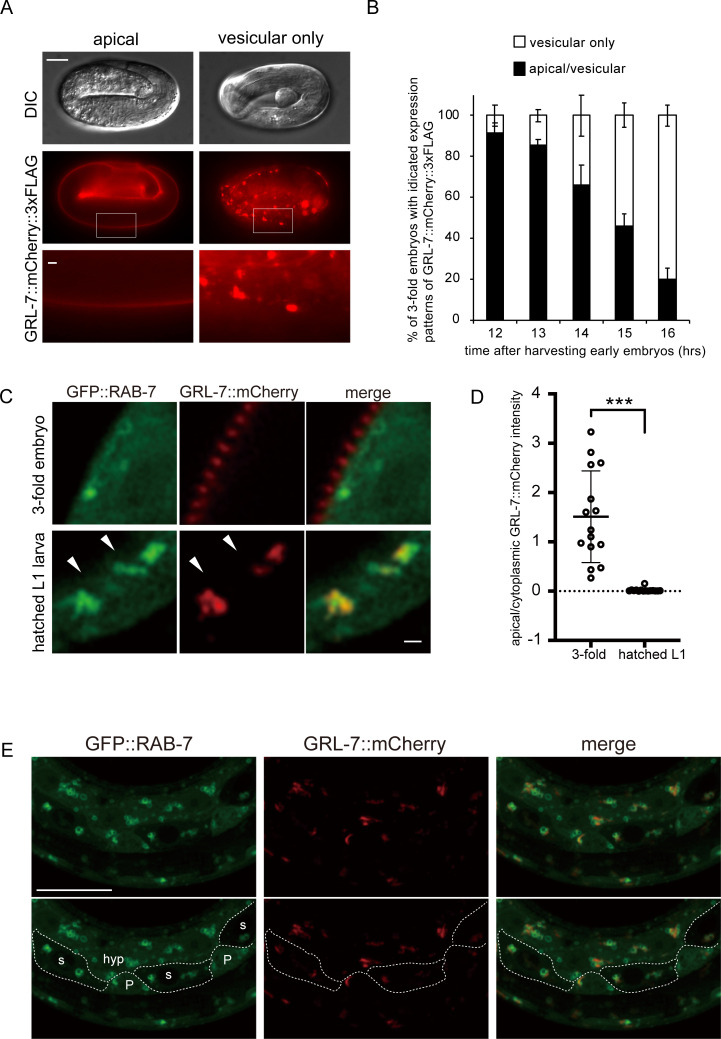
GRL-7 reporter proteins show expression patterns similar to that of PTR-18::GFP. (A) Expression patterns of GRL-7::mCherry::3xFLAG in the 3-fold embryos. GRL-7::mCherry::3xFLAG was observed at the apical side of the surface cells that cover the whole body (left panels; see S4 Fig for details) and/or vesicular structures (right panels) in the 3-fold embryos. The bottom panels are magnified views of the part of images in the middle (indicated by the rectangle). Scale Bar: 10 μm (whole-embryo image) and 1 μm (magnified image). (B) Percentage of 3-fold embryos exhibiting indicated localization of GRL-7::mCherry::3xFLAG. Data are presented as mean ± SD, and experiments were repeated three times. n ≥35 animals were scored for each experiment. (C) Super-resolution confocal images of animals expressing GRL-7::mCherry and GFP::RAB-7. GFP::RAB-7 overexpressed from the heterologous promoter distribute not only on late endosomes but also in the cytoplasm (see also Fig 5C). Arrowheads indicate the apical border of hypodermal cells. 3-fold embryos and hatched L1 larvae used for C and D were prepared 10 and 16 h after harvesting early embryos from gravid mothers, respectively. Scale bar: 1 μm. (D) Quantitation of GRL-7::mCherry fluorescent intensity. Apical and cytoplasmic GRL-7::mCherry were determined using the GFP::RAB-7 as a cytoplasm marker. 15 animals were scored for each. ***: P<0.001 (unpaired two-sided t-test). (E) Maximum intensity Z projection images showing a part of a newly hatched L1 larva expressing GFP::RAB-7 and GRL-7::mCherry used for C and D. GFP::RAB-7 expressed under the control of the *dpy-7* promoter visualizes the characteristic cell patterns of the borders (indicated by dotted lines at the bottom panels) of hypodermal (hyp 7), seam, and P cells, which are labeled as hyp, s, and P, respectively, in the image (refer to wormatlas; https://www.wormatlas.org/hermaphrodite/hypodermis/Hypframeset.html). Note that GRL-7::mCherry positive vesicle are found in all of these cells. Scale bar: 10 μm.

Although the loss of *grl-5*, *grl-7*, and *grl-27* can suppress the defects in *ptr-18* mutant animals, whether PTR-18 and these GRL proteins act in spatial and temporal proximity remains undetermined. To assess the interaction between *ptr-18* and *grl* genes, we constructed a *grl-7*::*mcherry*::*3xflag* gene by inserting the *mcherry*::*3xflag* tag into the *grl-7* genomic region in the fosmid WRM0615cE01 (see Materials and Methods). Similar to PTR-18::GFP, GRL-7::mCherry::3xFLAG was first detected in the 3-fold embryos (Fig 3A). Previous studies have shown that *grl-7* encodes a protein with a predicted signal sequence at the N-terminus, and its transcriptional reporter genes are expressed in hypodermal, seam and P cells [44]. Consistently, GRL-7::mCherry::3xFLAG localized along the apical side of the surface cells that cover the whole body as well as in the intracellular structures of these cells (Fig 3A; also see S4 Fig for details). As observed for PTR-18::GFP, the majority of the GRL-7::mCherry::3xFLAG embryos initially showed an apical distribution. However, as the embryos neared hatching, internal, vesicular localization became predominant (Figs 3B and S3A). After hatching under the feeding condition, apical localization of GRL-7::mCherry::3xFLAG remained undetectable until 11 h post-feeding of the L1-arrested larvae (S3B Fig). To further define the expression patterns of GRL-7, the *mcherry* tag was introduced before the stop codon of the *grl-7* gene using the CRISPR/Cas9 system, and its expression patterns were analyzed via super-resolution confocal microscopy. We first confirmed that GRL-7::mCherry was removed from the apical side of the cell before hatching by comparing its fluorescence intensity along the apical side of the cell and inside the cell, which is marked by cytoplasmically localized GFP::RAB-7, (Fig 3C and 3D; also see below). As predicted by the presence of N-terminal signal sequence [9], the apically localized GRL-7::mCherry does not overlap with cytoplasmic GFP::RAB-7, suggesting that GRL-7 was secreted (Fig 3C). During this analysis, we noticed that GRL-7::mCherry-positive vesicular structures were found in hypodermal, seam, and P cells in newly hatched larvae (Fig 3E). To compare the spatiotemporal dynamics of PTR-18 and GRL-7, the *ptr-18*::*gfp* fosmid reporter gene was introduced as an extrachromosomal array to the CRISPR-generated *grl-7*::*mcherry* strain (S4 Fig). GRL-7::mCherry exhibits striped patterns of localization, which likely shows annular furrows [50] (S4A, S4B and S4D Fig). On the other hand, PTR-18::GFP is relatively uniformly distributed over the body surface, consisting of hypodermal, seam, and P cells. Consistent with the prediction that PTR-18 localizes to the plasma membrane, PTR-18::GFP formed a layer underneath the apically distributed GRL-7::mCherry (S4C Fig). All of the embryos that expressed detectable levels of PTR-18::GFP also clearly showed apically localized GRL-7::mCherry (n = 25), consistent with the observations that the former was removed from the apical side slightly earlier than the latter (compare Figs 2B and 3B). We noticed that the PTR-18::GFP positive and GRL-7::mCherry positive internal structures show a partial overlap (S4E Fig). The co-localization was further confirmed via structured illumination microscopy in L4 larvae (S4F Fig).

Additionally, GRL-5::mCherry::3xFLAG expressed from the fosmid-based reporter gene showed a spatiotemporal expression pattern similar to that of GRL-7::mCherry::3xFLAG (S3C and S3D Fig). On the other hand, we could not detect the expression of GRL-27::mCherry::3xFLAG in several lines of animals carrying the *grl-27*::*mcherry*::*3xflag* fosmid-based transgene under the fluorescent microscope. In contrast to the GRL-7 and GRL-5 reporter proteins, mCherry-fused DPY-7 collagen protein remained predominant along the apical surface of hypodermal cells around hatching (S3E Fig). Previous studies have shown that DPY-7 protein localizes to annular furrows [51], at which GRL-7::mCherry also likely resides (S4 Fig). These observations implicated that dynamic remodeling of the cuticle components around the hatching takes place despite the absence of molting.

### PTR-18 and GRL-7 reporter proteins are internalized by endocytosis

**Fig 4:**
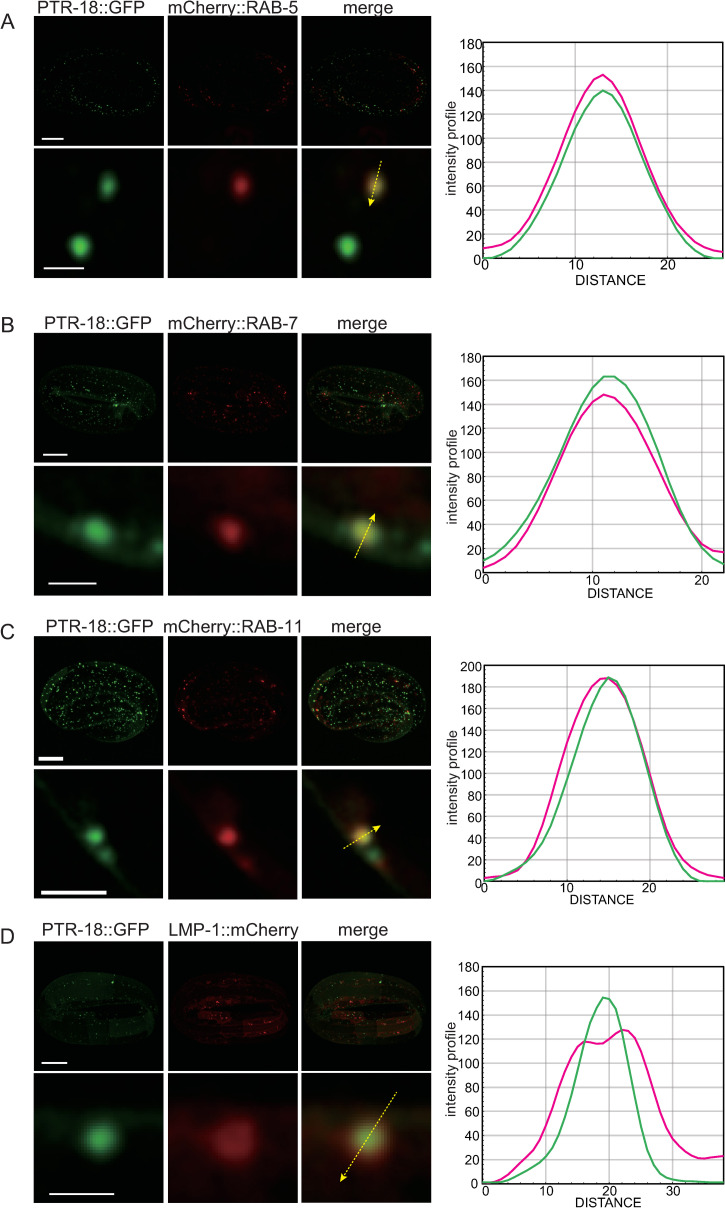
PTR-18::GFP localized to endosomes and lysosomes. Super-resolution images showing expression patterns of PTR-18::GFP and markers for endosomes and lysosomes. (A–D) Images on top rows show the maximum intensity Z projection of an entire embryo expressing indicated reporters, and scale bars indicate 10 μm. Images in the bottom rows show a magnified view of a Z section selected from the Z stacks of the above row, and scale bars indicate 1 μm (A, B, and D) or 2 μm (C). Intensity profiles along the arrow in each of the magnified image are shown on the right.

**Fig 5:**
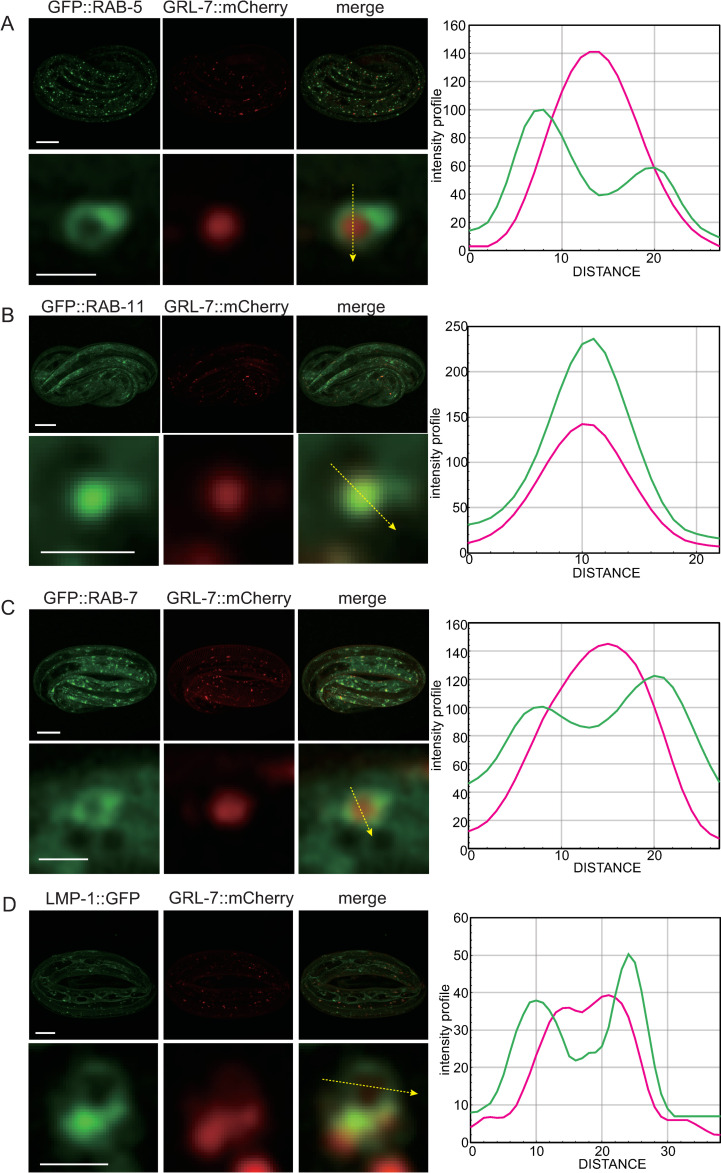
GRL-7::mCherry are localized to endosomes and lysosomes. Super-resolution images showing the expression patterns of GRL-7::mCherry and markers for endosomes and lysosomes. (A–D) Images on top rows show the maximum intensity Z projection of an entire embryo expressing indicated reporters, and scale bars indicate 10 μm. Images on bottom rows show a magnified view of a Z section selected from the Z stacks of the above row, and scale bars indicate 1 μm. Intensity profiles along the arrow in each of the magnified image are shown on the right.

To determine the identity of vesicles in the PTR-18::GFP internal structures, we transformed both *ptr-18*::*gfp* reporter and endo-lysosomal markers and analyzed them for potential co-localization by the super-resolution microscopy. PTR-18::GFP partially co-localized with mCherry::RAB-5 [52] (Fig 4A), mCherry::RAB-7 [53] (Fig 4B), mCherry::RAB-11 [53] (Fig 4C), and LMP-1::mCherry [54] (Fig 4D), which reside in early, late, and recycling endosomes and lysosomes, respectively. These observations suggest that PTR-18 is internalized by endocytosis, and a fraction can be recycled to the plasma membrane, while the other fraction is degraded in the lysosome. Next, we transformed the CRISPR-generated *grl-7*::*mcherry* strain with endo-lysosomal markers. Although most of the endosomes and lysosomes during the 3-fold stage could not be recognized as ring structures (Fig 4), GRL-7::mCherry localized within GFP::RAB-5, GFP::RAB-7, and LMP-1::GFP-positive vesicles (Fig 5A, 5C and 5D). In addition, GRL-7::mCherry colocalized with GFP::RAB-11 (Fig 5B). These findings suggest that the localization of both PTR-18 and GRL-7 is regulated by the endocytic pathway.

**Fig 6:**
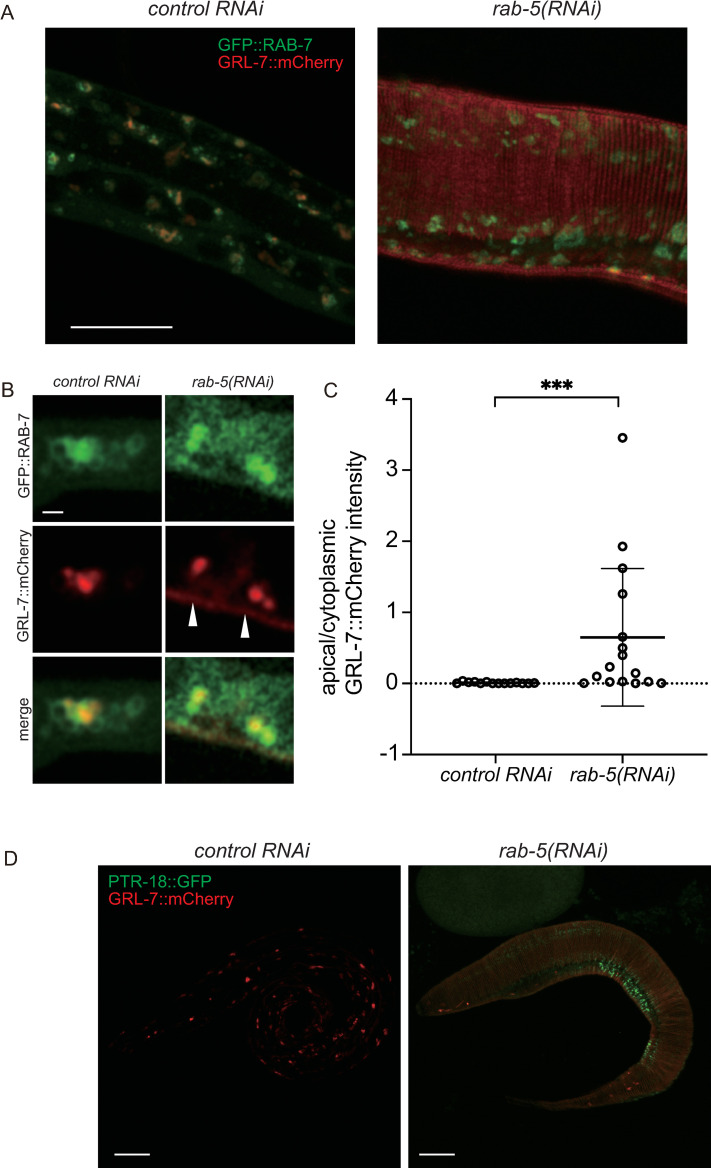
GRL-7::mCherry and PTR-18::GFP are internalized via endocytosis. (A) Images of maximum intensity Z projections showing a part of the bodies of newly hatched larvae. Note the apical localization of GRL-7::mCherry in the *rab-5(RNAi)* animal. Scale bar: 10 μm. (B) Magnified images of RNAi-treated animals. Apically localized GRL-7::mCherry are indicated by arrowheads. Scale bar: 1 μm. (C) Quantitation of fluorescence intensity of apical and cytoplasmic GRL-7::mCherry. Localization of apical and cytoplasmic GRL-7::mCherry are defined by the area outside and within GFP::RAB-7 distribution, respectively. ***: P<0.001 (unpaired two-sided t-test). (D) Apical localization of PTR-18::GFP are detected in newly hatched *rab-5(RNAi)* larvae. Images of maximum intensity Z projections are shown. Scale bar: 10 μm.

To test this possibility, we examined whether *rab-5(RNAi)*, which blocks endocytosis [52], suppresses the internalization of GRL-7::mCherry. Strikingly, some of the newly hatched *rab-5(RNAi)* L1 larvae exhibited apical, striped patterns of GRL-7::mCherry (Fig 6A). Quantitation of the fluorescence intensity of apical and cytoplasmic GRL-7::mCherry in newly hatched larvae indicates that *rab-5(RNAi)* interferes with the internalization of the reporter protein (Fig 6B and 6C). We also conducted *rab-5(RNAi)* using the CRISPR-generated *grl-7*::*mcherry* strains carrying *ptr-18*::*gfp* in extrachromosomal arrays. Whereas no newly hatched larvae derived from mothers treated with control RNAi showed detectable levels of PTR-18::GFP (n = 300), *rab-5(RNAi)* caused the apical distribution of PTR-18::GFP (Fig 6D; 18,9%; n = 53). These findings suggest that extracellular GRL-7 is sequestered with PTR-18 from the apical side of cells via endocytosis.

### *ptr-18* is required for the temporally-regulated internalization of GRL-7 reporter protein

**Fig 7:**
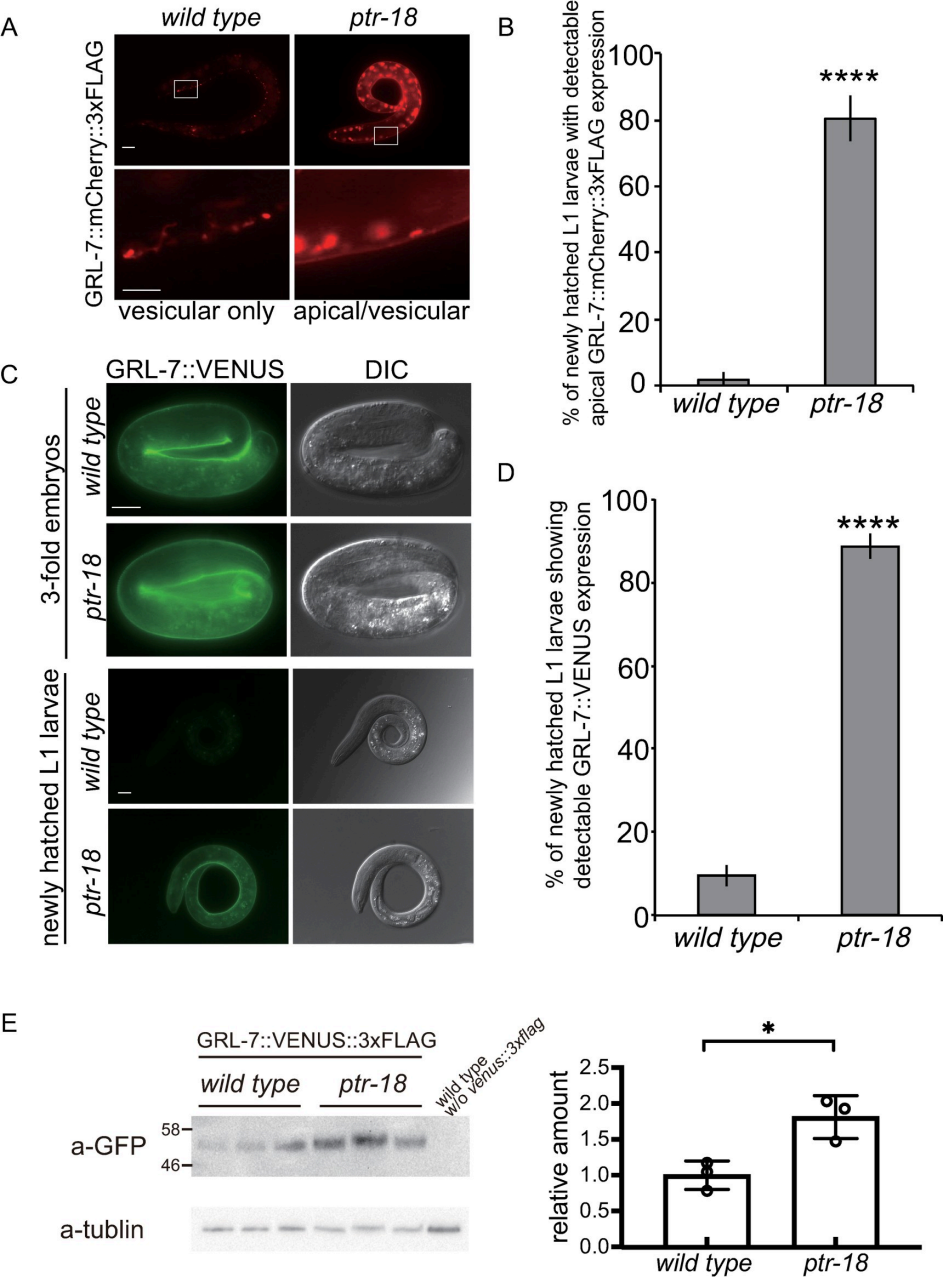
*ptr-18* is required for the timely internalization of GRL-7. (A) Newly hatched wild-type and *ptr-18* mutant L1 larvae showing vesicular (left) and apical/vesicular (right) distribution of GRL-7::mCherry::3xFLAG, respectively. The bottom panels are magnified views of the area within the rectangle from the images above. Scale bars indicate 10 μm (upper panel) and 1 μm (bottom panel). Photographs were taken with the same exposure time. (B) Apical localization of GRL-7::mCherry::3xFLAG was detected in most of the starved *ptr-18* mutant larvae. n ≥50 animals were scored for each genotype. Data were collected after 24 h L1 starvation and are presented as mean ± SD. Experiments were repeated three times, and n ≥50 animals were scored for each trial. ****: p <0.0001 (Fisher’s exact test). (C) Loss of *ptr-18* blocks the sequestration of GRL-7::VENUS. Wild-type and *ptr-18* mutant 3-fold embryos and L1 larvae after 24 h L1 starvation are shown. Photographs were taken with the same exposure time. Scale Bars: 10 μm. (D) Percentage of starved wild-type and *ptr-18* mutant L1 larvae showing GRL-7::VENUS expression. Data were collected after 24 h L1 starvation and are presented as mean ± SD. Experiments were repeated three times, and n ≥50 animals were scored for each trial. ****: p <0.0001 (Fisher’s exact test). (E) GRL-7 reporter protein is upregulated in newly hatched *ptr-18* mutant animals. Lysates from three biological samples were examined for newly hatched wild-type and *ptr-18* mutant larvae. GRL-7::VENUS::3xFLAG was detected by anti-GFP antibody. The specificity of the antibody was confirmed using the lysate from non-transgenic wild-type animals on the right lane. Intensity of GFP bands were normalized by those of the corresponding tubulin bands for quantitation. *: P <0.05 (unpaired two-sided t-test).

Genetic interactions suggest that *ptr-18* acts upstream of *grl-5*, *grl-7*, and *grl-27*. To test whether *ptr-18* contributes to the spatiotemporal distribution of these GRL proteins, a *grl-7*::*mcherry*::*3xflag* fosmid reporter was introduced into the *ptr-18* mutant animals. As shown in Fig 3A–3D, almost all of the newly hatched wild-type larvae showed a vesicular pattern of GRL-7::mCherry::3xFLAG localization (Fig 7A and 7B). In contrast, in the newly hatched *ptr-18* mutant larvae, GRL-7::mCherry::3xFLAG still accumulated along the apical surface of hypodermal cells (Fig 7A and 7B). These apically-localized GRL-7::mCherry::3xFLAG in the newly hatched *ptr-18* mutant larvae was eventually internalized, when these larvae were continuously cultured under starved and fed conditions (S5A–S5C Fig). This prolonged apical localization of GRL-7::mCherry::3xFLAG in starved *ptr-18* L1 larvae was suppressed by the expression of *ptr-18*::*venus* under the control of the P cell-specific, *hlh-3* promoter (S5D and S5E Fig), showing a correlation between defects in the clearance of apical GRL-7 reporter and maintenance of P cell quiescence (Fig 2E).

In general, GFP and its derivatives are sensitive to acid quenching in lysosomes. In contrast, RFP and its derivatives are relatively acid tolerant and resistant to lysosomal enzymes [55]. Similar to mCherry-fused GRL-7 reporter proteins, GRL-7::VENUS driven by the native promoter was found to exhibit apical localization in wild-type 3-fold embryos (Fig 7C). However, GRL-7::VENUS was undetectable after hatching (Fig 7D). These observations are consistent with the above data, suggesting that GRL-7 is internalized via endocytosis before hatching (Figs 5 and 6). Furthermore, most of the newly hatched *ptr-18* L1 larvae exhibited detectable levels of GRL-7::VENUS (Fig 7C and 7D), suggesting that the loss of *ptr-18* causes a significant delay in GRL-7 endocytosis. Although the difference in these expression patterns between wild-type and *ptr-18* mutant animals is obvious, it remains possible that this was caused by the overexpression of the *grl-7* reporter from extrachromosomal arrays. To exclude this possibility and obtain more quantitative insights into the regulation of GRL-7 protein levels by *ptr-18*, we introduced a *venus*::*3xflag* tag in front of the stop codon of the *grl-7* gene using the CRISPR/Cas9 system. Although the GRL-7::VENUS::3xFLAG expression was too faint for live cell imaging, immunoblot analysis showed that the level of tagged GRL-7 protein was upregulated in newly hatched *ptr-18* L1 animals compared to that in wild-type animals (Fig 7E). Thus, these observations explain why the loss of *ptr-18* causes developmental defects in a *grl-7*-dependent manner under the post-hatch starved condition, although neither PTR-18 nor GRL-7 protein was detected in newly hatched *wild-type* larvae. The untimely extracellular presence of GRL-7 due to the absence of PTR-18 will lead to the re-activation of the P cell irrespective of the dietary environment.

In contrast, the temporally controlled internalization of GRL-5::mCherry::3xFLAG was not affected by the loss of *ptr-18* (S6A and S6B Fig). Similar to GRL-7::VENUS, GRL-5::VENUS was also detected along the apical surface of hypodermal cells in both wild-type and *ptr-18* mutant 3-fold embryos (S6C Fig). However, GRL-5::VENUS was undetectable in both wild-type and *ptr-18* mutant L1 larvae (S6C and S6D Fig). Although we cannot exclude the possibility that similar to GRL-7, *ptr-18* also promotes timely internalization of GRL-5, the above observations suggest that PTR-18 dependent uptake is not the major route, if at all.

Previous studies have shown that both *daf-16/foxo* and *mir-235* mutant larvae fail to maintain the quiescence of multiple progenitor cells [2,6]. The temporally regulated internalization of GRL-7::mCherry::3xFLAG was not affected in newly hatched *daf-16/foxo* and *mir-235* L1 larvae, suggesting that these genes and *ptr-18* regulate L1 arrest through distinct mechanisms (S7A Fig). *mir-235* downregulates *grl-7* via the miR-235 target site on the 3’UTR of *grl-7* mRNA [8]. In contrast, our findings suggest that *ptr-18* suppresses *grl-7* via endocytosis-mediated degradation, raising the possibility that *ptr-18* and *mir-235* regulate the quiescence of P cells in genetically parallel pathways. Consistent with this, the loss of *mir-235* in *ptr-18* mutant animals significantly enhanced the quiescent defective phenotype (S7B Fig).

These findings suggest that the internalization and subsequent lysosomal degradation of GRL-7 before hatching play critical roles in establishing the capacity of neural progenitor cells to maintain quiescence under starved conditions.

### The GXXXDD and GXXXD/E motifs and C-terminal cytoplasmic region of PTR-18 protein are required for appropriate localization and function

**Fig 8:**
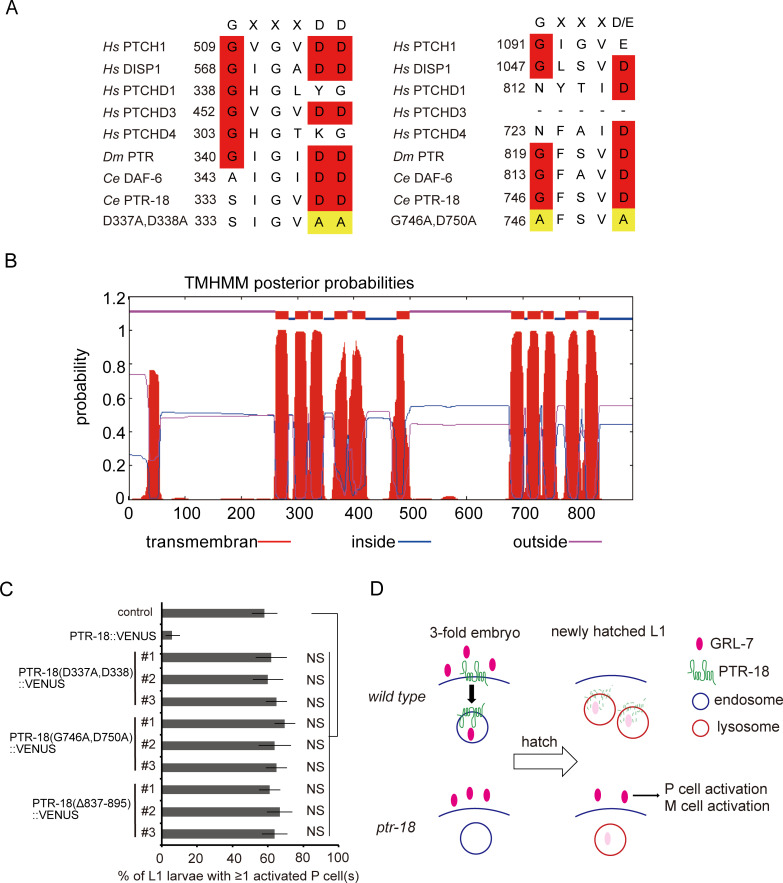
The GXXXDD and GXXXD/E motifs and C-terminal cytoplasmic region of PTR-18 protein are required for appropriate function. (A) Comparison of GXXXDD and GXXXD/E motifs. The amino acid substitutions introduced in PTR-18(D337A, D338A)::VENUS and PTR-18(G746A, D750A)::VENUS are shown in the bottom panel. (B) Membrane topology of PTR-18 predicted by the TMHMM algorithm. Note that the C-terminal portion of PTR-18 is predicted to be cytoplasmic. (C) Effects of mutated PTR-18::VENUS on the defects of *ptr-18* mutant animals in maintaining P cell quiescence. Animals that did not inherit the array expressing PTR-18::VENUS were used as “control.” NS: Not statistically significant (Fisher’s exact test). (D) Model of the clearance of extracellular GRL-7 by PTR-18. In 3-fold, wild-type embryos, PTR-18 promotes the endocytosis of secreted GRL-7 to facilitate their degradation, so that newly hatched animals can retain developmental quiescence under a starvation condition. In contrast, residual activity of extracellular GRL-7 in newly hatched, *ptr-18* mutant animals can cause unwanted initiation of L1 developmental events such as P and M cell activation when the animals happen to encounter a starvation condition. Although PTR-18 and GRL-7 are transported together to lysosomes, whether PTR-18 and GRL-7 physically associate with each other remains undetermined.

Similar to the Patched and Dispatched proteins of the Hh signaling pathway, PTR/PTCHD contains GXXXDD and GXXXD/E motifs within TM4 and TM10, respectively [13]. These proteins belong to the RND transporter superfamily [21], and the GXXXD motif in its bacterial prototype transporters is indispensable for their transporter activity [23,24]. Furthermore, mutations in GXXXDD and GXXXD/E motifs have been shown to be functionally essential for Patched and Dispatched proteins [13,25–27]. To test whether these conserved motifs (Fig 8A) and carboxy-terminal cytoplasmic tail, which is predicted by the TMHMM algorithm (Fig 8B) [56], critically contributed to the activity of PTR-18, PTR-18(D337A, D338A)::VENUS, PTR-18(G746A, D750A)::VENUS, and PTR-18(Δ837–895)::VENUS were expressed in *ptr-18(ok3532)* animals. All these mutant PTR-18::VENUS proteins failed to restore the defect in maintaining P cell quiescence (Fig 8C). These findings demonstrated that GXXXDD and GXXXD/E motifs and putative carboxy-terminal cytoplasmic tail are required for appropriate function. Therefore, PTR-18 is most likely a transporter, similar to the bacterial RND transporters, human PTCH, and *C*. *elegans* PTC-3 [25,57–59].

Altogether, we conclude that *ptr-18* temporally restricts the activity of GRL-7 within 3-fold stage of embryogenesis by facilitating its internalization and subsequent lysosomal degradation. Defects in this temporal restriction cause prolonged accumulation of extracellular GRL-7 beyond hatching, which compromises the capacity of neural progenitor cells to respond to nutritional stress (Fig 8D).

## Discussion

### *ptr-18*-dependent restriction of GRL activity allows neural progenitor cells to anticipate nutritional stresses before hatching

In this study, we first found that one of the *C*. *elegans ptr/ptchd* orthologs, *ptr-18*, is required to prevent P neuronal progenitor cells from undergoing unwanted reactivation when newly hatched larvae encounter starvation conditions. This reactivation requires the activity of *grl-5*, *grl-7*, and *grl-27 hh*-r genes but not other *grl* genes known to be expressed in the hypodermal and P cells. While PTR-18 first begin to accumulate along the apical cell membrane of hypodermal, seam and P cells at the late embryonic stage, GRL-7 becomes enriched at the specific regions of the cuticle, which are probably annular furrows. PTR-18 is subsequently internalized slightly earlier than GRL-7. However, both proteins eventually together populate endosomal and lysosomal compartments, resulting in the clearance of their apically localized fractions before hatching. Loss of *ptr-18* activity causes significant delay in this endocytosis-mediated degradation of GRL-7, such that newly hatched *ptr-18* mutant larvae still exhibit extracellular GRL-7 accumulation. Furthermore, the potential transporter activity of PTR-18 is required for its appropriate function. These findings suggest that *ptr-18* temporally limits the activity of GRL-7 to establish the capacity of neural progenitor cells to maintain quiescence in response to nutritional stresses and also provide unique insights into the cellular role of PTR/PTCHD in promoting the clearance of extracellular Hh-related protein by targeting it to lysosomal degradation.

### Potential role of PTR-18 as a decoy receptor for GRL-7

Our studies suggest that PTR-18 temporally restricts the availability of extracellular GRL-7 protein by targeting it to lysosomal degradation via endocytosis. Similarly, previous studies have shown that Hh receptor Patched sequesters Hh through endocytosis to limit the spatiotemporal range of its action in *Drosophila* and vertebrates [60–63]. In addition to sequestering Hh ligand, Patched also confers “ligand-independent antagonism” against Hh signaling by inhibiting Smo activity [60]. On the other hand, the reactivation of P cells observed, caused by the loss of *ptr-18*, was almost completely dependent on the activity of *grl* genes. Thus, in contrast to Patched, PTR-18 is unlikely to be coupled to the signaling components that act downstream of GRL-7. PTR-18 seems to act as its decoy receptor, whose function is only to sequester extracellular GRL-7 protein. This model is reminiscent of the D6 chemokine receptor, which has been proposed to act as a decoy receptor that is unfit for signaling, but can scavenge chemokines by constitutively delivering it to lysosomes [64,65] and whose loss results in prolonged inflammation due to impaired chemokine clearance [66]. This could explain the observation that the expression of *ptr-18* in hypodermal or P cells can restore the quiescent defective phenotype of *prt-18* mutant animals (Fig 2E). In these experiments, *ptr-18* could be overexpressed under the control of the heterologous promoter from the extrachromosomal arrays, and when sufficient amount of PTR-18 protein is expressed in either type of cells, extracellular GRL-7 protein would be removed before hatching.

In addition to PTR-18, other PTR/PTCHD proteins such as *Drosophila* PTR and the *C*. *elegans* DAF-6 reporter protein reportedly localize to unidentified intracellular vesicles [36,37,67]. Conversely, *daf-6* mutations result in excessive accumulation of glial-secreted extracellular matrix in a channel formed by glial cells [68], which led to the model where *daf-6* antagonizes the secretion of vesicles containing the matrix or promotes their uptake [69]. Although the involvement of *hh*-r genes in *daf-6*-dependent processes remains unexplored, reporters of some *hh*-r genes have been shown to be expressed in the glial socket and sheath cells, in which *daf-6* restricts the channel size [9,38,44,70]. In contrast to the *hh*-r genes [9,44], a comprehensive reporter expression analysis of *C*. *elegans ptr* genes has not yet been reported. However, a genome-wide expression analysis showed that most of the *ptr* genes exhibit oscillatory expression patterns, similarly to *hh*-r genes [71]. Considering that *C*. *elegans hh*-r and *ptr* genes are extensively diverged [9,11,72], each PTR protein may further fine-tune the oscillatory activity of a particular set of Hh-r proteins via their endocytosis-mediated degradation.

### Receptor for GRL-5, GRL-7, and GRL-27

In this study, we showed that *ptr-18* acts upstream of the *grl-5*, *grl-7*, and *grl-27* genes. Little is known about the genetic pathway that the *hh*-r genes act on. In *Drosophila* and vertebrates, Hh and sonic hedgehog (Shh) stimulate Hh signaling through their receptor, Patched [73,74]. Although *C*. *elegans* possesses two genes, *ptc-1* and *ptc-3*, which encode patched orthologs, their relationship with *hh*-r genes remains ambiguous. However, RNAi targeting *ptc-3* and several *ptr* and *hh*-r genes results in molting defects [12], suggesting that *ptc-3* and at least some of the *ptr* and *hh*-r genes may participate in similar pathways [10]. Our attempt to test whether *ptc-3* is involved in maintaining L1 arrest was hampered by the severe embryonic lethality caused by the loss of *ptc-3*. This technical difficulty will need to be overcome for further elucidation of the *ptr-18*-dependent regulation of L1 arrest.

Do PTR/PTCHD proteins in other animals also act as decoys for Hh? Overexpression of human PTR/PTCHD, PTCHD1, and PTCH53 (also called PTCHD4) proteins in Shh-responsive C3H10T1/2 cells suppressed Hh signaling [29,75]. In contrast, the expression of PTCHD1 in *patched1* (*ptch1*)-deficient mouse embryonic fibroblasts did not repress the canonical Hh signaling pathway [76]. These observations raise the possibility that PTCHD functions upstream of Patched, possibly by acting as a decoy receptor for Shh. However, the expression of PTCH53 in the DAOY cancer cell line inhibited the upregulation of the Hh pathway via the Smo agonist, purmorphamine, raising the possibility that PTCH53 acts downstream of Smo [75]. In contrast to these *in vitro* studies, there is insufficient evidence that *Drosophila* PTR and mammalian PTCHD proteins impinge upon the Hh pathway *in vivo*. Unlike *ptch1*-deficient mice, *ptchd1*-knockout mice did not show overproliferation of neuronal precursors in the brain [77]. In vertebrates, a vertebrate-specific Hh-interacting protein 1 (Hhip1, also called Hip1) also functions together with Patched to antagonize Hh activity through its direct binding [78–82]. Thus, the loss of *ptchd1* might be compensated by *Hhip1* in vertebrates. In *Drosophila*, which does not have the *Hhip1* ortholog, Hh is still internalized in the absence of Patched [63]. Thus, this patched-independent internalization might be mediated by PTR.

### *hh*-r genes may promote the progression of larval development

We have shown that *grl-5*, *grl-7*, and *grl-27* contribute to the reactivation of quiescent P cells in starved *ptr-18* L1 larvae. Although we could not detect the expression of the *grl-27* fosmid reporter gene, we observed the accumulation of extracellular GRL-5 and GRL-7 reporter proteins only prior to hatching and around L1 molting but not around the time when P cells initiate ventral migration. On the other hand, simultaneous loss of *grl-5*, *grl-7*, and *grl-27* did not cause a significant delay in P cell reactivation in well-fed L1 larvae. These observations raise the possibility that there is an additional Hh-r protein that is induced by feeding earlier than GRL-5, GRL-7, and GRL-27 and activates P cell migration. How can ectopic activation of these *grl* genes cause P cell reactivation and later developmental events in starved *ptr-18* and *mir-235* mutant larvae? Starved *ptr-18* and *mir-235* animals occasionally molt. On the other hand, previous studies have shown that most *ptr* and *hh*-r genes show transcriptional oscillations with distinct phases during larval stages and that multiple *hh*-r and *ptr* genes as well as *ptc-3* are implicated in molting [12,71,83]. Thus, molting observed in starved *ptr-18* and *mir-235* mutant larvae is likely caused by the ectopic activation of transcriptional oscillations of *hh*-r and *ptr* genes. Furthermore, the activation of at least two *hh*-r-dependent processes, P cell reactivation and molting, always occur in this order. This apparent dependency of the latter events on the former can be explained by assuming 1) that each temporal transcriptional upregulation of *hh*-r genes promotes the subsequent *hh*-r genes one after another and 2) that each of the waves of upregulation sequentially activates distinct L1 developmental events. If so, the inhibition of *grl-7* in starved *ptr-18* and *mir-235* mutant larvae would block the upregulation of a *hh*-r gene that promotes P cell reactivation in fed wild-type larvae by preventing the ectopic initiation of the transcriptional oscillations. This idea would also explain why *ptr-18* deficiency conspicuously affected only the sequestration of the GRL-7 reporter but GRL-5 reporter. *grl-5*, potentially as well as *grl-27*, might act upstream of *grl-7* to promote its oscillation of expression. Thus, the loss of *grl-5* and potentially *grl-27* would significantly suppress the quiescent defective phenotype of *ptr-18* mutant animals via the downregulation of *grl-7*. Although the molecular mechanism that generates the transcriptional oscillations of *hh*-r and *ptr* genes remain to be fully elucidated, it is worth noting that *nhr-23*, which encodes a nuclear hormone receptor homologous to both mammalian RORα and *Drosophila* DHR3, has been shown to upregulate multiple *hh*-r and *ptr* genes, including *grl*-5, *grl*-7, and *ptr-18*, and regulate molting [84–86]. In addition to *nhr-23*, dozens of genes whose inhibition causes molting defects have already been identified [87]. Instead of just regulating the molting cycle, some of these genes may promote the sequential activation of multiple developmental events by controlling the transcriptional oscillations of *hh*-r and *ptr* genes.

## Materials and methods

Further information and requests for resources and reagents should be directed to and will be fulfilled by the Lead Contact, Masamitsu Fukuyama (mfukuyam@mol.f.u-tokyo.ac.jp).

### *C*. *elegans* strains

All nematode strains were cultured according to standard procedures [88]. The strains used in this study are listed below. The following strains were purchased from the *Caenorhabditis* Genetics Center: RB2058 *grl-2(ok2721)V*, RB1097 *grl-4(ok1076)IV*, RB2018 *grl-5(ok2671) V*, RB1999 *grl-7(ok2644) V*, RB2495 *grl-15(ok3455) III*, RB2322 *grl-17(ok3017) V*, RB2112 *grl-21(ok2791) IV*, PK172 *ptc-1(ok122) unc-4(e120)/mnC1 dpy-10(e128) unc-52(e444) II*, VC851 *ptr-2(ok1338)/szT1 [lon-2(e678)] I*, *+/szT1 X*, VC20514 *ptr-3(gk333566) II*, VC1110 *+/szT1[lon-2(e678)] I*, *ptr-4(ok1576)/szT1 X*, VC1067 *ptr-5(gk472) X*, VC2301 *ptr-6(ok2988) II*, MY1, which possesses the polymorphism WBVar01902623(S787Ochre) in the *ptr-8* locus, RB1693 *ptr-10(ok2106) I*, VC20409 *ptr-11(gk100342) I*, VC40042 *ptr-12(gk105502) I*, VC40161 *ptr-17(gk490736) I*, RB2542 *ptr-18(ok3532) II*, RB2393 *ptr-20(ok3263) II*, VC3219 *ptr-23(ok3663) I*, and PD4666 *ayIs6 X*. Further, the following strain was purchased from the National BioResource Project: FX10640 *grl-27(tm10640) IV*. RB2058, RB1097, RB2018, RB1999, RB2495, RB2322, RB2112, and FX10640 were outcrossed against N2 six times and designated as YB3786, YB3788, YB3790, YB3289, YB3847, YB3848, YB3793 and YB4020, respectively, prior to experiments, while RB2542 was outcrossed against N2 four times to yield YB2891. *grl-10(td193)* was constructed by replacing the protein coding region of the *grl-10* gene with Self-Excising Drug Selection Cassette (SEC), as described in [89], using a repair template plasmid, pCH104.1 and pCH102.1 and pCH103.1, both of which encode sgRNAs targeting the *grl-10* locus. The introduced SEC was excised from the genome by heat shock, resulting in the *td193* allele. pCH102.1 was made by PCR using primers, MF1515 and CH346 and PU6::unc-119_sgRNA plasmid as a template [90]. Similarly, pCH103.1 was made using primers, MF1515 and CH347. pCH104.1 was constructed by inserting homology arms derived from PCR products made with primer pairs CH342/CH343 and CH344/CH345 into pDD282 [89].

### Construction of plasmids, and transformation of *C*. *elegans*

Fosmid-based reporter genes for *ptr-18*, *grl-5*, *grl-7* and *gel-27* were constructed using the fosmids WRM0613dH03, WRM0631aE09, WRM0615cE01 and WRM0636cB11, respectively, using “recombineering” as described previously [91]. GFP and *mCherry*::*3x flag* tags were PCR-amplified using pBalu1 and pCH78.1 plasmids, respectively. The primers used are listed in the Key Resources Table 1. Transformation of *C*. *elegans* was performed as described previously [92].

**Table 1:** Key Resources Table.

**Plasmids**		
pBalu1	[91]	
pBalu4	[91]	
pMF435.1	[101]	
pMF449.1	[7]	
pMF450.1	[6]	
pMF674.1	[6]	
pMF826.1	*Pptr-18*::*ptr-18 cDNA*::*venus*::*unc-54* 3’UTR	
pKM189.1	P*ptr-18*::*mcherry*::*unc-54* 3’UTR	
pMF843.2	*ptr-18*::*gfp* reporter: *gfp* was inserted into WRM0613dH03.1	
pMF875.1	P*dpy-7*::*mcherry*::*rab-11* cDNA:: *unc-54* 3’UTR	
pCH1.1	P*hlh-3*::*ptr-18* cDNA::*venus*::*unc-54* 3’UTR	
pCH15.1	P*lin-48*::*ptr-18* cDNA::*venus*::*unc-54* 3’UTR	
pCH27.1	P*dct-5*::*ptr-18* cDNA::*venus*::*unc-54* 3’UTR	
pCH29.1	P*dpy-7*::*ptr-18* cDNA::*venus*::*unc-54* 3’UTR	
pCH51.1	5×QUAS::*ptr-18* cDNA::*venus*::*unc-54* 3’UTR	
pCH78.1	*3xflag* tag was inserted after *mcherry* cDNA in pBalu4	
pCH83.1	*mcherry*::*3×flag* tag derived from pCH78.1 was inserted into the *grl-7* genomic region in the fosmid WRM0615cE01	
pCH92.1	P*dpy-7*::*gfp*::*rab-5* cDNA::*unc-54* 3’UTR	
pCH93.1	P*dpy-7*::*mcherry*::*rab-5* cDNA::*unc-54* 3’UTR	
pCH94.1	P*dpy-7*::*gfp*::*rab-5* cDNA::*unc-54* 3’UTR	
pCH98.1	P*dpy-7*::*gfp*::*rab-7* cDNA::*unc-54* 3’UTR	
pCH99.1	P*dpy-7*::*mcherry*::*rab-7* cDNA::*unc-54* 3’UTR	
pCH101.1	P*dpy-7*::*lmp-1* cDNA::*mcherry*::*unc-54* 3’UTR	
pCH102.1	P*U6*::*grl-10_sgRNA*	
pCH103.1	P*U6*::*grl-10_sgRNA*	
pCH104.1	Repair template used to generate *grl-10(td193)*	
pCH106.1	P*ptr-18*::*ptr-18* cDNA(D337A, D338A)::*venus*::*unc-54* 3’UTR	
pCH107.1	P*ptr-18*::*ptr-18* cDNA(G746A, D750A)::*venus*::*unc-54* 3’UTR	
pCH110.1	P*ptr-18*::*ptr-18* cDNA(Δ837–895)::*venus*::*unc-54* 3’UTR	
pCH111.1	*mcherry*::*3×flag* tag derived from pCH78.1 was inserted into the *grl-5* genomic region in the fosmid WRM0615cE01	
pCH118.1	*mcherry*::*3×flag* tag derived from pCH78.1 was inserted into the *grl-27* genomic region in the fosmid WRM0636cB11	
pCH121.1	P*grl-7*::*grl-7*::*venus*::*grl-7* 3’UTR	
pCH126.1	P*grl-5*::*grl-5*::*venus*::*grl-5* 3’UTR	
pCH130.1	P*dpy-7*::*dpy-7 cDNA*::*mcherry*::*unc-54* 3’UTR	
**Strains**		
CF1038	*daf-16(mu86)I*	
MY1	*ptr-8(WBVar01902623) II*	
RB1693	*ptr-10(ok2106) I*	
RB2393	*ptr-20(ok3263) II*	
VC1067	*ptr-5(gk472) X*	
VC1110	*+/szT1[lon-2(e678)] I; ptr-4(ok1576)/szT1 X*	
VC20409	*ptr-11(gk100342) I*	
VC20514	*ptr-3(gk333566) II*	
VC3219	*ptr-23(ok3663) I*	
VC40042	*ptr-12(gk105502) I*	
VC40161	*ptr-17(gk490736) I*	
VC851	*ptr-2(ok1338)/szT1 [lon-2(e678)] I; +/szT1 X*	
YB1812	*ayIs6 X*	
YB2890	*ptr-6(ok2988) II*	
YB2891	*ptr-18(ok3532) II*	
YB3028	*ptr-18(ok3532) II; ayIs6 X*	
YB3092	*ptr-18(ok3532) II; tdEx2021[pMF826*.*1(100 ng/μl) + pMF435*.*1(50 ng/μl) +pRF4 (50 ng/μl)]*	
YB3093	*ptr-18(ok3532) II; tdEx2022[pMF826*.*1(100 ng/μl) + pMF435*.*1(50ng/μl) +pRF4 (50ng/μl)]*	
YB3094	*ptr-18(ok3532) II; tdEx2023[pMF826*.*1(100 ng/μl) + pMF435*.*1(50 ng/μl) +pRF4 (50 ng/μl)]*	
YB3127	*ptr-18(ok3532) II; tdEX2046[pCH1*.*1(100 ng/μl) + pMF435*.*1(50 ng/μl) + pRF4 (50 ng/μl)]*	
YB3128	*ptr-18(ok3532) II; tdEx2047[pCH1*.*1(100 ng/μl) + pMF435*.*1(50 ng/μl) + pRF4 (50 ng/μl)]*	
YB3129	*ptr-18(ok3532) II; tdEx2048[pCH1*.*1(100 ng/μl) + pMF435*.*1(50 ng/μl) + pRF4 (50 ng/μl)]*	
YB3131	*ptr-18(ok3532) II; tdEX2051[pCH15*.*1(100 ng/μl) + pMF435*.*1(50 ng/μl) + pRF4 (50 ng/μl)]*	
YB3132	*ptr-18(ok3532) II; tdEX2052[pCH15*.*1(100 ng/μl) + pMF435*.*1(50 ng/μl) + pRF4 (50 ng/μl)]*	
YB3133	*ptr-18(ok3532) II; tdEX2053[pCH15*.*1(100 ng/μl) + pMF435*.*1(50 ng/μl) + pRF4 (50 ng/μl)]*	
YB3231	*ptr-18(ok3532) II; tdEX2111[pCH29*.*1(1 ng/μl) + pMF435*.*1(50 ng/μl) + pRF4 (50 ng/μl)]*	
YB3232	*ptr-18(ok3532) II; tdEX2112[pCH29*.*1(1 ng/μl) + pMF435*.*1(50 ng/μl) + pRF4 (50 ng/μl)]*	
YB3233	*ptr-18(ok3532) II; tdEX2113[pCH29*.*1(1 ng/μl) + pMF435*.*1(50 ng/μl) + pRF4 (50 ng/μl)]*	
YB3287	*ptr-18(ok3532) II*	
YB3289	*grl-7(ok2644) V*	
YB3295	*ptr-18(ok3532) II;grl-7(ok2644) V*	
YB3317	*ptr-18(ok3532) II; tdEX2172[pHK497*.*1(1 ng/μL) + pHK497*.*1(10 ng/μL) + pCH51*.*1(1 ng/μL) + pKM66*.*1(25 ng/μL) + pRF4(50 ng/μL)]*	
YB3318	*ptr-18(ok3532) II; tdEX2173[pHK497*.*1(1 ng/μL) + pHK497*.*1(10 ng/μL) + pCH51*.*1(1 ng/μL) + pKM66*.*1(25 ng/μL) + pRF4(50 ng/μL)]*	
YB3319	*ptr-18(ok3532) II; tdEX2174[pHK497*.*1(1 ng/μL) + pHK497*.*1(10 ng/μL) + pCH51*.*1(1 ng/μL) + pKM66*.*1(25 ng/μL) + pRF4(50 ng/μL)]*	
YB3359	*tdEx2207[pKM189*.*1(25 ng/μl) + pMF843*.*2(125 ng/μl) + pRF4 (50 ng/μl)]*	
YB3586	*tdEx2259[pMF449*.*1(1 ng/μl) + pCH83*.*1(10 ng/μl) + pRF4(10 ng/μl)]*	
YB3640	*ptr-18(ok3532) II; tdEx2259[pMF449*.*1(1 ng/μl) + pCH83*.*1(10 ng/μl) + pRF4(10 ng/μl)]*	
YB3753	*mir-235(n4504) I; tdEx2259[pMF449*.*1(1 ng/μl) + pCH83*.*1(10 ng/μl) + pRF4(10 ng/μl)]*	
YB3754	*daf-16(mu86) III; tdEx2259[pMF449*.*1(1 ng/μl) + pCH83*.*1(10 ng/μl) + pRF4(10 ng/μl)]*	
YB3786	*grl-2(ok2721) V*	
YB3788	*grl-4(ok1076) IV*	
YB3790	*grl-5(ok2671) V*	
YB3793	*grl-21(ok2791) IV*	
YB3795	*ptr-18(ok3532) II; tdEx2269[pCH27*.*1(5 ng/μl) + pMF435*.*1(5 ng/μl) + pRF4(25 ng/μl)]*	
YB3803	*ptr-18(ok3532) II; tdEx2300[pMF450*.*1(10 ng/μl) + pRF4(50 ng/μl)]*	
YB3806	*ptr-18(ok3532) II; tdEx2269[pCH27*.*1(5 ng/μl) + pMF435*.*1(5 ng/μl) + pRF4(25 ng/μl)]*	
YB3807	*ptr-18(ok3532) II; tdEx2269[pCH27*.*1(5 ng/μl) + pMF435*.*1(5 ng/μl) + pRF4(25 ng/μl)]*	
YB3808	*tdEx2303[pMF843*.*2(10 ng/μl) + pCH99*.*1(1 ng/μl) + pRF4(50 ng/μl)]*	
YB3809	*tdIs54[pMF843*.*2(10 ng/μl) + pDP#MM51(50 ng/μl)]; tdEx2274[pCH83*.*1(1 ng/μl) + pRF4(100 ng/ μl)]*	
YB3820	*tdEx2314[pMF843*.*2(5 ng/μl) + pCH93*.*1(0*.*5 ng/μl) + pRF4(50 ng/μl)]*	
YB3847	*grl-15(ok3455) III*	
YB3848	*grl-17(ok3017) V*	
YB3851	*ptr-18(ok3532) II; grl-2(ok2721) V*	
YB3854	*ptr-18(ok3532) II; grl-4(ok1076) IV*	
YB3857	*ptr-18(ok3532) II; grl-5(ok2671) V*	
YB3858	*ptr-18(ok3532) II; grl-15(ok3455) III*	
YB3862	*ptr-18(ok3532) II; grl-17(ok3017) V*	
YB3864	*ptr-18(ok3532) II; grl-21(ok2791) IV*	
YB3896	*tdEx2347[pMF843*.*2(10 ng/μl) + pCH101*.*1(1 ng/μl) + pRF4(50 ng/μl)]*	
YB3897	*tdEx2347[pMF843*.*2(10 ng/μl) + pMF875*.*1(1 ng/μl) + pRF4(50 ng/μl)]*	
YB3949	*tdEx2388[pMF826*.*1(20 ng/μl) + pMF435*.*1(20 ng/μl) + pRF4(10 ng/μl)]*	
YB3954	*tdEx2394[pCH106*.*1(20 ng/μl) + pMF435*.*1(20 ng/μl) + pRF4(10 ng/μl)]*	
YB3957	*tedEx2397[pCH107*.*1(20 ng/μl) + pMF435*.*1(20 ng/μl) + pRF4(10 ng/μl)]*	
YB3967	*tdEx2406[pCH110*.*1(20 ng/μl) + pMF435*.*1(20 ng/μl) + pRF4(10 ng/μl)]*	
YB4020	*grl-27(tm10640) V*	
YB4021	*ptr-18(ok3532) II; grl-27(tm10640) V*	
YB4022	*ptr-18(ok3532) II; grl-10(td193) V*	
YB4028	*grl-10(td193) V*	
YB4029	*tdEx2245[pMF449*.*1(1 ng/μl) + pCH111*.*1(10 ng/μl) + pRF4(10 ng/μl)]*	
YB4053	*ptr-18(ok3532) II; tdEx2441[pMF826*.*1(20 ng/μl) + pMF435*.*1(20 ng/μl) + pRF4(10 ng/μl)]*	
YB4057	*ptr-18(ok3532) II; tdEx2445[pCH106*.*1(20 ng/μl) + pMF435*.*1(20 ng/μl) + pRF4(10 ng/μl)]*	
YB4058	*ptr-18(ok3532) II; tedEx2446[pCH107*.*1(20 ng/μl) + pMF435*.*1(20 ng/μl) + pRF4(10 ng/μl)]*	
YB4143	*ptr-18(ok3532) II; tdEx2393[pCH106*.*1(20 ng/μl) + pMF435*.*1(20 ng/μl) + pRF4(10 ng/μl)]*	
YB4144	*ptr-18(ok3532) II; tdEx2394[pCH106*.*1(20 ng/μl) + pMF435*.*1(20 ng/μl) + pRF4(10 ng/μl)]*	
YB4145	*ptr-18(ok3532) II; tedEx2396[pCH107*.*1(20 ng/μl) + pMF435*.*1(20 ng/μl) + pRF4(10 ng/μl)]*	
YB4146	*ptr-18(ok3532) II; tedEx2397[pCH107*.*1(20 ng/μl) + pMF435*.*1(20 ng/μl) + pRF4(10 ng/μl)]*	
YB4147	*ptr-18(ok3532) II; tdEx2405[pCH110*.*1(20 ng/μl) + pMF435*.*1(20 ng/μl) + pRF4(10 ng/μl)]*	
YB4148	*ptr-18(ok3532) II; tdEx2406[pCH110*.*1(20 ng/μl) + pMF435*.*1(20 ng/μl) + pRF4(10 ng/μl)]*	
YB4149	*ptr-18(ok3532) II; tdEx2407[pCH110*.*1(20 ng/μl) + pMF435*.*1(20 ng/μl) + pRF4(10 ng/μl)]*	
YB4313	*tdEx2561[pMF450*.*1(1 ng/μl) + pCH121*.*1(10 ng/μl) + pRF4(50 ng/μl)]*	
YB4335	*ptr-18(ok3532) II; tdEx2245[pMF449*.*1(1 ng/μl) + pCH111*.*1(10 ng/μl) + pRF4(10 ng/μl)]*	
YB4336	*grl-5(ok2671) V; grl-7(ok2644) V; grl-27(tm10640) V*	
YB4339	*ptr-18(ok3532) II; tdEx2561[pMF450*.*1(1 ng/μl) + pCH121*.*1(10 ng/μl) + pRF4(50 ng/μl)]*	
YB4359	*ptr-18(ok3532) II; tdEX2046[pCH1*.*1(100 ng/μl) + pMF435*.*1(50 ng/μl) + pRF4 (50 ng/μl)]; tdIs56[pCH83*.*1(1 ng/μl) + pRF4(100 ng/ μl)]*	
YB4360	*ptr-18(ok3532) II; tdEX2047[pCH1*.*1(100 ng/μl) + pMF435*.*1(50 ng/μl) + pRF4 (50 ng/μl)]; tdIs56[pCH83*.*1(1 ng/μl) + pRF4(100 ng/ μl)]*	
YB4361	*ptr-18(ok3532) II; tdEX2048[pCH1*.*1(100 ng/μl) + pMF435*.*1(50 ng/μl) + pRF4 (50 ng/μl)]; tdIs56[pCH83*.*1(1 ng/μl) + pRF4(100 ng/ μl)]*	
YB4376	*tdEx2569[pCH126*.*1(10ng/μl) + pMF450*.*1(1ng/μl) + pRF4(50ng/μl)*	
YB4454	*ptr-18(ok3532) II; tdEx2569[pCH126*.*1(10ng/μl) + pMF450*.*1(1ng/μl) + pRF4(50ng/μl)*	
YB4456	*tdEx2612[pCH130*.*1(1 ng/μl) + pCH121*.*1(1 ng/μl) + pRF4(50 ng/μl)]*	
YB4662	*mir-235(n4504)I;ptr-18(ok3532) II*	
YB4663	*grl-7(td256)V*: Crispr-generated *grl-7*::*venus*::*3xflag* strain	
YB4668	*grl-7(td261)V*: Crispr-generated *grl-7*::*mcherry* strain	
YB4671	*ptr-18(ok3532) II; grl-7(td256)V*	
YB4706	*grl-7(td261)V*:*tdEx2746[pMF843*.*2(10 ng/μl)* + *pBS SK+(50 ng/μl) + pRF4(40 ng/μl)]*	
YB4718	*grl-7(td261)V*:*tdEx2751[pCH98*.*1* + *pBS SK+(*60 *ng/μl) + pRF4(40 ng/μl)]*	
YB4722	*grl-7(td261)V*:*tdEx2751[pCH92*.*1* + *pBS SK+(*60 *ng/μl) + pRF4(40 ng/μl)]*	
YB4724	*grl-7(td261)V*:*tdEx2753[pCH94*.*1* + *pBS SK+(*60 *ng/μl) + pRF4(40 ng/μl)]*	
YB4726	*grl-7(td261)V*:*tdEx2755[pCH100*.*1* + *pBS SK+(*60 *ng/μl) + pRF4(40 ng/μl)]*	
**Primers used for plasmid construction**		
*lin-48* promoter	CH50	ATCCTGCAGCCTGCATTTTTTTCAGAGTC
	CH51	ATCCCCGGGCTGAAATTGAGCAGAGCTGAA
*dct-5* promoter	CH83	ATCCTGCAGTTTCAACGGGAATTGAACTTTTG
	CH84	ATCCCCGGGTATATATGGGTCCCAACTTTCAA
*mcherry*::*3x flag for grl-7 reporter*	CH264	ACTGCCAAGAGACCAAAGGGGATATCTCATGCTACACCTACCGTCAATTGATGGTCTCAAAGGGTGAAGAAGATAAC
	CH265	TTTCTATATTGCACTTGGAACACTGTTTTTGAAATCTTTGATTTCTATATTTACTTGTCATCGTCATCCTTG
*mcherry*::*3x flag for grl-5 reporter*	CH422	GTGAGACTGAGAAGGACGGAACCACGTGCTTCGCTTTCAAACAATCTTCTATGGTCTCAAAGGGTGAAGAAGATA
	CH423	TTAAAGGTATTTGAATAAGAAATTATTAGTTCAAAAGTACACCAGTCTTCTTACTTGTCATCGTCATCCTTGTAA
P*ptr-18*::*ptr-18* cDNA(D337A, D338A)::*venus*::*unc-54* 3’UTR	CH400	GCAGCTGTGTTCATTTTTATTCACG
	CH401	TACCCCGATCGACAAAATTAAGAAGGGA
P*ptr-18*::*ptr-18* cDNA(G746A, D750A)::*venus*::*unc-54* 3’UTR	CH402	GCATTCTCTGTAGCTTTTGTAGCACATATTACGT
	CH403	GATGGCCATGAGAAGTGTGG
P*ptr-18*::*ptr-18* cDNA(Δ837–895)::*venus*::*unc-54* 3’UTR	CH408	GCTAGTATGGTGAGCAAGGG
	CH409	TGTGAATGGGAGGGCTGAGA
*rab-5* cDNA	CH300	ATCACTAGTATGGCCGCCCGAAACGCAGGAACCGC
	CH301	ATCGCTAGCTTTACAGCATGAACCCTTTTGTTGCT
*rab-7* cDNA	CH348	ATCACTAGTATGTCGGGAACCAGAAAGAAGGCGCT
	CH349	ATCACTAGTACAATTGCATCCCGAATTCTGCTGGT
*rab-11* cDNA	MF1995	ACGTACTAGTATGGGCTCTCGTGACGATGAATACG
	MF1996	GCATGCTAGCTGGGATGCAACACTGCTTCTTTGGT
*lmp-1* cDNA	CH350	ATCCCCGGGATGTTGAAATCGTTTGTCATCTTGTT
	CH351	ATCACCGGTGACGCTGGCATATCCTTGTCTCTTTG
*venus* cDNA	MF1432	GCACTAGTATGGTGAGCAAGGGCGAGGAGC
	MF1433	GACGCTAGC CTTGTACAGCTCGTCCATGCCG
*ptr-18* promoter	KM342	GGGGAACTAGTGTGAATGGGTGATTG
	KM339	ACCCCCGGGGTCAAGGCTCAGGCTCAATCAACTGC
*ptr-18* cDNA	KM340	TCCCCCGGGATGAAATCAATATCACAATGTCTTGGCAAC
	KM341	ACCCCCGGGGCTAGCAGCCCGCTCAGCGCTCCGAACGGAT
*ptr-18* recombineering	MF1813	CAACAAAAGAGGAACCAGTTGAAAAATCCGTTCGGAGCGCTGAGCGGGCTATGAGTAAAGGAGAAGAACTTTTCA
	MF1814	GGAAAAAAATTTAAAAAATAAATAAAAAATTACGGAAAACAAAAAAGTTATTTGTATAGTTCATCCATGCCATG
*grl-5* ORF	CH482	GCTAGCCATGCCCGGGATGAGAACTGCGGTGTTG
	CH483	GCCCTTGCTCACCATAGAAGATTGTTTGAAAGCG
*mcherry*::*3x flag for grl-27 reporter*	CH440	CCTACTGTGGAGCCTCAAAGAATGGGCACAACTGTCACGCCTTTGCAATGATGGTCTCAAAGGGTGAAGAAGATA
	CH441	TTTCTGACAAGGTTCAAAATATACAGACACCAAATTAATTGCATGCATCACTTGTCATCGTCATCCTTGTAATCG
Introducing 3xFlag tag into pBalu4	CH262	CATGACATCGATTACAAGGATGACGATGACAAGTAAAAGGGCGAATTCCAGCACACTGGCGG
	CH263	ATCTTTATAATCACCGTCATGGTCTTTGTAGTCCTTATACAATTCATCCATGCCACCTG
pCH102.1	MF1515	AAACATTTAGATTTGCAATTCAATT
	CH346	GCAATGAGTGTCTCATTTTTTGTTTTAGAGCTAGAAATAGCAA
pCH103.1	MF1515	As above
	CH347	GCAATGAGTGTCTCATTTTTTGTTTTAGAGCTAGAAATAGCAA
pCH104.1	CH342	ATCGGGCCCGAGGCGCGGTCTCGATTAAGAAAATT
	CH343	ATCGTCGACTTTTTAGGTGAAGTGGGTCGTGTTTC
	CH344	ATCCCCGGGATTAATCGCTTCTTGCTTGTACATTT
	CH345	ATCACTAGTGCCGCTACTGCCTCGATTTTCTTTTC
**Primers used for detecting alleles of the indicated mutant strains**		
*ptr-18(ok3532)II*	KM328	ATTTGCCGAAGTTGCATAGG
	KM329	TTCACAAAATGCGACCATCT
	KM330	AAATCACATTTTTCGGAGCTT
	KM331	TGAAGAATTCTGGCAAATATCG
	KM391	CAGGCACTCAGTGCGCGACC
	KM394	AGTGGGAGAAGTGGCGAGTCG
*grl-7(ok2644)V*	KM267	CTTTGAATGGCAGTGTGACG
	KM268	AGACCCTCCGTACCTGGTTT
	KM269	CTATTGCATAACCGCCCATC
	KM270	AAGCTCGGAAGCGTGCTG
	KM273	TGCTCAAACCACATCAGCAT
	KM274	GAACTTGAGCTGGAGCAACC
*grl-2(ok2721)V*	CH312	GGCTCCTCCACCTCTTATCC
	CH313	ATTTTCTATCGCCTGGCCTT
	CH314	GCTCCAGTATCTGCTCCATCA
	CH315	CTTTTTCACAACCGGGAATC
	CH316	CCACCAATGCCAATTGTGAC
	CH317	GGTGCCATTCCTTCCAGACT
*grl-4(ok1076)IV*	MF1802	AGTGATCAGAGATGGGCTGG
	MF1803	AAGCCACGTAACAAAATCCG
	MF1804	GGCAATGTCGAGAAGGAAAC
	MF1805	TACTGTCCAGGGGAGATTCG
	MF1815	GAGGTAATGGAGGCGGTGCC
	MF1816	GGGCTTCCACATCTGCAGCA
*grl-5(ok2671)V*	CH318	TGTAAATGTTGTTCCACCGC
	CH319	TTTTTCCCTCTTTATCCCGC
	CH320	AAACTTTCTGCAAAATGCTCTACA
	CH321	CCAGCCCAACCTAAGCCTAA
	CH322	CAGCACAGCAATCATCCTTC
	CH323	TGTTTGAAAGCGAAGCACGTGG
*grl-10(td193)V*	CH416	CCAACATCCATCCCTCATTC
	CH417	ACAATGCCAAGAGACGCCAC
	CH418	GAGGAGAGAAACACGACCCA
	CH419	TCTAAGCTGCTGATCGGTCA
	CH420	AGAGCTGCTGCATTTGGAGT
	CH421	ACCCTCAGCTGCTAGGTTGA
*grl-15(ok3455)III*	CH324	AGCGAAAGTCTCGAGAAACG
	CH325	AAGCCGTCAAAGTGGTGAAA
	CH326	CCACAAAGAGGCGGAGTCTA
	CH327	CACCTTTTTCATGATTGCCA
	CH328	AGGTGCCTTGGATTCTTGGA
	CH329	GTGCTACAGTGTCACTACAG
*grl-17(ok3017)V*	CH330	CTACGTTCAAAGCGGAGGAG
	CH331	TGATTGGCACATAGTCGGAA
	CH332	GAAAAACATGGCAACCTTCC
	CH333	AAATGACAGATTGAAGCGGG
	CH334	GGAGGTGGAGGTAAGGCTTC
	CH335	TTGTGCTGCGATGTCTTCTC
*grl-21(ok2791)IV*	MF1798	TCTGAAGGTTCTAGGGGGTC
	MF1799	CTGAATCGGAAGAAGCGAGG
	MF1800	TCCTGCGCAATGACGCGACG
	MF1801	CGAATGGCTGCTTTGTGCAG
	MF1809	GTTACAGCACTGGCGGTCAC
	MF1810	GACTTGGCTCTGTATGGTCC
*grl-27(tm10640)V*	CH336	TATAGCCCTCCGATTTGTTC
	CH337	AATGGTTCCGCCAAGCGCT
	CH338	AACGCCCTTCAGAACATCAC
	CH339	TGCACATCACATCCTGGTCT
	CH340	ATGAGAAGGGAGGATTGCA
	CH341	CGTGGTGAGACCATTTCGCA
**Primers used for *grl-7* reporter strains by the CRISPR/Cas9 system**		
*venus*::*3xflag with homologous arms*	MF2984	GACCGGTCATAACTATTCATTCAATAGGTTAACATTTAATGTTGCAGCTATGTGACCAACACCGAGCTCTACTGCCAAGAGACCAAAGGGGATATCTCATGCTACACCTACCGTCAATTGATGGTGAGCAAGGGCGAGGAGCTGT
	MF2985	AATACTTTATTATTAGTCGAACCAGGAAATAATATAACAAAGAGGAGAAGAAAATGAAACAATTTCCTTTCTATATTGCACTTGGAACACTGTTTTTGAAATCTTTGATTTCTATATTTACTTATCGTCATCGTCTTTATAGTCA
*venus*	MF2607	ATGGTGAGCAAGGGCGAGGAGCTG
	MF2608	CTTGTACAGCTCGTCCATGCCGAGAG
*mcherry with homologous arms*	MF3407	GTGGCGTAATTTTACGACCGGTCATAACTATTCATTCAATAGGTTAACATTTAATGTTGCAGCTATGTGACCAACACCGAGCTCTACTGCCAAGAGACCAAAGGGGATATCTCATGCTACACCTACCGTCAATTGATGGTGAGCAAGGGCGAGGAGGATA
	MF3408	CTGTTTATTAAGCGAATACTTTATTATTAGTCGAACCAGGAAATAATATAACAAAGAGGAGAAGAAAATGAAACAATTTCCTTTCTATATTGCACTTGGAACACTGTTTTTGAAATCTTTGATTTCTATATTTACTTGTACAGCTCGTCCATGCCGCCGG
*mcherry*	MF3049	ATGGTGAGCAAGGGCGAGGAGGATA
	MF3050	TTACTTGTACAGCTCGTCCATGCCGCCGG

### Construction of *grl-7* reporter genes using the CRISPR/Cas9 system

*venus*::*3xflag* and *mCherry* tags were inserted into the *grl-7* gene using the CRISPR/Cas9 system according to the protocol described in [93]. Briefly, *venus*::*3xflag* and *mCherry* tags, both of which contain *grl-7* homologous arm sequences at both the 5’ and 3’ ends were constructed via PCR using primer pairs MF2984-MF2985 and MF3047-MF3048, respectively. DNA fragments encoding *venus* and *mCherry* cDNA were PCR-amplified using primer pairs MF2607-MF2608 and MF3049-MF3050, respectively. These PCR products were subsequently annealed to make “dsDNA hybrid donors”, as described in [93]. Each of these dsDNA hybrid donors was co-injected with Alt-R S.p. Cas9 Nuclease V3 (IDT, 1081058), Alt-R CRISPR-Cas9 tracrRNA (IDT, 1072532), gene-specific Alt-R CRISPR-Cas9 crRNA, and pRF4 at certain concentrations as suggested by [93]. The partial sequence of the *grl-7* gene CTATATTTACAATTGACGGT was used to custom-order the *grl-7*-specific Alt-R CRISPR-Cas9 crRNA. On the other hand, we could not insert *venus* and HA tags using CRISPR-Cas9 crRNAs targeting to the sequences, CTCCGAACGGATTTTTCAAC, AACCAGTTGAAAAATCCGTT, and CCGTTCACTTTGATAACTTT in the *ptr-18* locus.

### Immunostaining

A drop of the starved L1 larvae was placed on polylysine-coated slides, permeabilized using the freeze-cracking method, and fixed in MeOH/acetone at -20°C for 5 min, as described previously [94]. The slides were incubated with anti-PGL-1 antibody [95] diluted 1:40,000 in PBS containing 4% BSA at 4°C overnight. The slides were then washed in PBS three times and incubated with Alexa Fluor 568 goat anti-mouse IgG (H+L) highly cross-adsorbed secondary antibody (Thermo Fisher Scientific) diluted 1:2,000 in PBS containing 4% BSA at 37°C for 30 min. The slides were washed in PBS three times and subsequently mounted with PermaFluor mounting medium (Thermo Fisher Scientific) containing 1 μg/mL of DAPI (4’,6-diamidino-2-phenylindole).

### Microscopy

*The C*. *elegans* embryos and adults were mounted on 4% agar pads, as described previously [96]. Fluorescent and differential interference contrast (DIC) images were obtained with an Axio Imager M1 equipped with Plan-Apochromat 63x/1.40 Oil DIC and HRm digital camera, and processed with the AxioVision (Carl Zeiss) and Photoshop (Adobe) software.

### Structured illumination microscopy

*C*. *elegans* was cultured at 20°C in NGM agar plates. Live worms were immobilized with 50 mM levamisole in M9 and mounted on a slide with 2% agarose. The worms were imaged with a DeltaVision OMX Optical Microscope (version 4), Software: DeltaVision OMX softWoRx. Oil 1.518. Objective 60X NA 1.42. Pixel size: x = 0.08, y = 0.08, and z = 0.125 μm. All images were reconstructed using the same parameters. Images were post-processed with OMERO.web 5.3.4-ice36-b69.

### Super-resolution microscopy

L1 larvae were anesthetized as described above. Super-resolution imaging was performed using a Zeiss LSM980 quipped with Airysccan2 and the Plan-Apochromat 63x/Oil 1.4 DIC objective. Approximately 150 Z-section images/embryo were acquired using the SR-4Y Multiplex mode at 0.17 μm intervals and processed with ZEN pro software and Fiji.

### *C*. *elegans* culture

The L1 larvae were prepared and either starved in complete S medium or grown synchronously as described previously [7]. The concentration of L1 larvae was adjusted by culturing sterilized embryos at 10 embryos/μl. The starved L1 larvae in polypropylene tubes were continuously rotated at 30–40 rpm. The L1 larvae referred to as ‘after 5 days of L1 starvation’ indicate larvae that were allowed to hatch and were cultured in complete S medium for 5 days after the alkali/bleach treatment, except for L1 larvae in Figs 6A–6D and S6A–S6D, which were starved in cholesterol- and ethanol-free complete S medium for 24 h.

The synchronized embryos shown in Figs 2B and 3B and S2A and S3A were prepared by harvesting the early embryos by bleaching gravid adults as described above, hatched, and cultured in cholesterol- and ethanol-free complete S medium in a 15 mL polypropylene tube with continuous rotation at 30–40 rpm and 20°C. Embryos were observed 9–17 h after bleach treatment. Embryos in other experiments were harvested in M9 buffer using a spatula and a glass Pasteur pipet (after washing out the well-fed gravid worms) on 100 mm 4x peptone plates, which contained a 4x peptone concentration of the standard nematode growth medium (NGM) agar. The collected embryos were then washed five times with M9 buffer to remove *E*. *coli* before observation.

### Immunoblot analysis

Wild-type and *ptr-18* mutant animals after 24 h L1 starvation were prepared as described in *C*. *elegans culture*. Newly hatched larvae were first filtered through an L1 harvest filter (InVivo Biosystems, USA). Filtered animals were collected via centrifugation in 15 mL polypropylene tubes at 3,500 rpm for 1 min at 4°C, further centrifuged in 1.5 mL microtubes at 15,000 rpm for 1 min at 4°C, and snap-frozen by liquid nitrogen. Proteins were extracted in the urea lysis buffer (6 M urea, 2 M thiourea, 3% [w/v] CHAPS, 1% [v/v] Triton X- 100; [97]) by sonication using an ultrasonic disruptor UD-100 equipped with a TP-120 tip (TOMY, Japan). UD-100 was set to repeat the cycle of 10 s pulses at 99% power and 10 s intervals for 2 min. When unbroken larvae were found under the dissecting microscope, another around of sonication was performed. Protein extracts were resolved in 10% TGX by SDS-PAGE and blotted onto the Immun-blot PVDF membrane by the Transblot Turbo blotting system (Bio-Rad, USA). Blotted membranes were first blocked in the Everyblot blocking buffer for 5 min (Bio-Rad, USA), incubated with primary antibodies at 4°C overnight, and subsequently incubated with secondary antibodies at room temperature (20–25°C) for 1 h. Anti-GFP from mouse IgG1κ (clones 7.1 and 13.1) (Roche 11814460001), anti-α-tubulin antibody, mouse monoclonal clone DM1A (Sigma T6199), and Peroxidase-AffiniPure Goat Anti-Mouse IgG (Jackson ImmunoResearch 115-035-071) were used at 1:1,000, 5, 000, and 10,000 dilutions, respectively, in a 19:1 Tris Buffered Saline:Blocking One (Nacalai, Japan) solution containing 1% Tween 20. Signals were detected using Chemi-lumi One (Nacalai, Japan) with ChemiDoc XRS+ (Bio-Rad, USA). The gray intensity of each band circled by a fixed size of ROI was measured using Fiji (https://imagej.net/Fiji).

### Viability assay

The embryos harvested using the alkali/bleach method described above and the resulting hatched larvae were cultured at 10 embryos/μl in 10 mL of cholesterol-, ethanol-free complete S medium in a 15 mL polypropylene tube with rotation at 30–40 rpm at 20°C for 10 days. The larvae were subsequently transferred onto freshly seeded plates, and the number of transferred and recovered larvae was scored after 3 days.

### Feeding RNAi

Feeding RNAi targeting *rab-5* was conducted using the ORFeome-RNAi v1.1 library [98] according to the standard procedure [99]. Empty pPD129.36 (L4440) vector [100] was used as a negative control for the RNAi experiments. L4 larvae expressing PTR-18::GFP and GRL-7::mCherry were transferred onto the RNAi plates, and the starved L1 larvae were prepared as previously described.

### Statistical analysis

All statistical analyses were conducted using the Microsoft Excel software.

## Supporting information

S1 Fig Loss of *ptr-18* does not affect the quiescence of primordial germ cells and L1 survival. (A) Primordial germ cells, Z2 and Z3 (arrows), were visualized using anti-PGL-1 antibody. The absence of the proliferation of Z2 and Z3 was scored under the Nomarski microscopy after 5-day starvation in complete S media. Experiments were repeated three times, and n ≥35 animals were scored for each trial (see text). Scale bar: 10 μm. (B) Newly hatched L1 larvae were starved in cholesterol/EtOH-free complete S medium, and the viability was assessed after 10 days of culture. Experiments were repeated three times, and n ≥50 animals were scored for each trial. Data are presented as mean ± SD. ****: p <0.0001 (Fisher’s exact test). (C) Simultaneous loss of *grl-5*, *grl-7*, and *grl-27* does not cause the delay of the timing of P cell activation. Data are presented as mean ± SD. Experiments were repeated three times, and n ≥50 animals were scored for each trial. N.S.: Not statistically significant (Fisher’s exact test).
(PDF)

S2 Fig *ptr-18* acts in P and hypodermal cells. (A) Percentage of animals at each developmental stage after harvesting early embryos. Data are presented as mean ± SD. Experiments were repeated three times, and ≥50 animals were scored for each time point. (B) Hypodermal expression of PTR-18::GFP becomes detectable 11 h after the L1-arrested larvae are fed. Descendants of P cells (arrows) and F, K, and U cells (arrowhead) are indicated. Scale Bar: 10 μm. (C) Seam cells with or without PTR-18::GFP expression are shown in red and white arrowheads, respectively. The arrow indicates hypodermis. Scale Bar: 10 μm. (D) Expression of PTR-18::VENUS driven by the indicated promoters in 3-fold embryos. Expression of PTR-18::VENUS driven by the *dct-5* promoter was only detectable after hatching. The arrow and arrowhead in the left panels indicate the excretory duct and hypodermal cells, respectively. Arrowheads in other panels point to cells indicated below. Types of cells expressing each reporter gene are indicated below each image. Scale Bar: 10 μm. (E) Expression of PTR-18::VENUS and mCherry driven by the *hlh-3* promoter. P cells are indicated by arrowheads. Scale Bar: 10 μm. (F) PTR-18::VENUS is expressed in hypodermal but not P cells when driven by the Q system. The constructs introduced are indicated on the left. Note that the expression of PTR-18::VENUS was downregulated below detectable levels in P cells (arrowheads). Scale Bar: 10 μm. (G) Schematic of the hypodermis-specific expression of PTR-18::VENUS. The *dpy-7* promoter drives the expression of transcriptional activator QF in both the hypodermal and P cells. However, QS expressed in P cells under the control of the *hlh-3* promoter suppresses QF, resulting in the expression of PTR-18::VENUS only in the hypodermal cells.
(PDF)

S3 Fig Expression patterns of *grl-7*, *grl-5*, and *dpy-7* reporter genes. (A) Percentages of 3-fold embryos and L1 larvae after harvesting early embryos via the bleach treatment of gravid adults. Data are presented as mean ± SD. Experiments were repeated three times, and n ≥50 animals were scored for each trial. (B) Expression patterns of GRL-7::mCherry::3xFLAG in fed L1 larvae. Images shown are of L1 larvae 1 h (top panels) and 11 h (bottom panels) after the L1-arrested larvae were transferred to the fed condition. The photographs were taken with the focal planes on the apical surface (apical) and the ventral midline (midline). GRL-7::mCherry::3xFLAG localized at the vesicular structure and along the apical membrane are indicated by the arrowhead and arrow, respectively. The right panels are magnified views of the areas within the rectangle in the images on the left. Scale Bar: 10 μm (DIC image) and 2 μm (magnified image). (C) Expression of GRL-5::mCherry::3xFLAG during embryogenesis. Differential interference contrast and fluorescent images of embryos carrying the *grl-5*::*mcherry*::*3xflag* reporter gene. Scale Bar: 10 μm. (D) Expression of GRL-5::mCherry::3xFLAG in fed L1 larvae. Photographs show fed L1 larvae at indicated hours after the L1-arrested larvae were fed. Note that GRL-5::mCherry::3xFLAG accumulates at the apical surface of the whole body 11 h after the transfer. Photographs at the bottom are magnified views of the part of the above ones (indicated by the rectangles). All photographs showing GRL-5::mCherry::3xFLAG expression were taken at the same exposure time for comparison. Scale Bar: 10 μm (DIC image) and 1 μm (magnified image). (E) Expression of DPY-7::mCherry in 3-fold embryos (top panels) and newly hatched L1 larvae (bottom panels). The photograph of the L1 larva was taken after 24 h L1 starvation. Scale Bar: 10 μm.
(PDF)

S4 Fig Coexpression of *ptr-18* and *grl-7* reporter genes. Super-resolution images showing the expression of CRISPR-generated *grl-7*::*mCherry* reporter gene co-expressed with *ptr-18*::*gfp* derived from a fosmid. Trios of images below show PTR-18::GFP (green), GRL-7::mCherry (red), and both, respectively, from left to right. (A) Maximum intensity Z-projection image of an embryo showing apical distributions of PTR-18::GFP and GRL-7::mCherry. Scale bar: 10 μm. (B) Maximum intensity Z-projection image of an embryo showing apical and vesicular distributions of PTR-18::GFP and apical localization of GRL-7::mCherry. (C) Magnified view from a Z section of an embryo indicated in B. Intensity profiles are determined along the arrow in the merged image. Scale bar: 10 μm. (D) Maximum intensity Z-projection image of an embryo showing predominantly vesicular patterns of PTR-18::GFP and apical and vesicular distributions of GRL-7::mCherry. Scale bar: 10 μm (upper panel) and 1 μl (bottom panel). (E) Magnified view from a Z section of an embryo indicated in B. Intensity profiles are determined along the arrows in the merged image. Note that PTR-18::GFP only localized to GRL-7::Cherry-positive vesicles at the left and middle regions, but not at the right. Intensity profiles are determined along the corresponding arrows in the merged image. Scale bars: 1 μm. (F) PTR-18 and GRL-7 localize to the same compartment. Structured illumination microscopy of L4 worms expressing PTR-18::GFP and GRL-7::mCherry. PTR-18 and GRL-17 populate the same compartment. While PTR-18 is present on the limiting membrane, GRL-7 accumulates inside. The nature of the compartment likely changes during development, comparing the top and bottom panels. N = 8 animals. Arrowheads point to the PTR-18 and GRL-17 positive compartments.
(PDF)

S5 Fig *ptr-18* promotes temporally controlled endocytosis of GRL-7. (A) GRL-7::mCherry::3xFLAG expression in starved wild-type and *ptr-18* L1 larvae. Bottom panels are magnified views of the part of the middle panels (indicated by the rectangles). Scale bars indicate 10 μm (upper panel) and 1 μm (bottom panel). (B) Percentage of animals showing apical GRL-7::mCherry::3xFLAG localization during L1 starvation. GRL-7::mCherry::3xFLAG localization was scored at the indicated time points of L1 starvation in complete S medium. Note that the presence of cholesterol and ethanol in the medium does not affect the phenotype after 24 h L1 starvation (compare to Fig 7A and 7B, where L1 larvae were scored after 24 h L1 starvation in cholesterol-, ethanol-free complete S medium). Data are presented as mean ± SD. Experiments were repeated three times, and n ≥50 animals were scored for each trial. ****: p <0.0001 (Fisher’s exact test). (C) Percentage of animals showing apical GRL-7::mCherry::3xFLAG localization under the fed condition. GRL-7::mCherry::3xFLAG localization was scored at the indicated time points after feeding L1-arrested animals. Data are presented as mean ± SD. Experiments were repeated three times, and n ≥50 animals were scored for each trial. *: P<0.05, ***: P <0.001, and ****: p <0.0001 (Fisher’s exact test). (D) Expression of *ptr-18*::*venus* in P cells can suppress the prolonged apical localization of GRL-7::mCherry::3xFLAG in starved *ptr-18(ok3532)* L1 larvae. *ptr-18* mutant larvae with or without the indicated transgenes after 24 h L1 starvation are shown. Scale Bars: 10 μm. (E) Percentage of newly hatched, *ptr-18(ok3532)* mutant larvae showing apical localization of GRL-7::mCherry::3xFLAG in the presence or absence of the transgenes that drives the expression of *ptr-18*::*venus* under the control of the P-cell specific *hlh-3* promoter. Data are presented as mean ± SD. Experiments were conducted with three transgenic lines (#1 to #3) and repeated three times. n ≥50 animals were scored for each trial. ****: p <0.0001 (Fisher’s exact test). Siblings of line #2 that did not inherit the transgenes were used as controls.
(PDF)

S6 Fig Loss of *ptr-18* does not significantly affect the timely internalization of GRL-5. (A) Both newly hatched wild-type and *ptr-18* mutant L1 larvae showing the vesicular distribution of GRL-5::mCherry::3xFLAG. The bottom panels are magnified views of the area within the rectangle from the images above. Scale bars indicate 10 μm (upper panel) and 1 μm (bottom panel). Photographs were taken with the same exposure time. B) Apical localization of GRL-5::mCherry::3xFLAG was rarely detected in the starved wild-type and *ptr-18* mutant L1 larvae. n ≥50 animals were scored for each genotype. Data were collected after 24 h L1 starvation and are presented as mean ± SD. Experiments were repeated three times, and n ≥50 animals were scored for each trial. (C) Wild-type and *ptr-18* mutant 3-fold embryos and L1 larvae after 24 h L1 starvation are shown. These animals carry transgenes that express GRL-5::VENUS fusion protein under the control of the native *grl-5* promoter. Note that the fluorescence of L1 animals under the GFP filter is derived from gut granules. The presence of the transgene is marked by the co-injected plasmid that expresses mCherry in the hypodermis. Photographs were taken with the same exposure time. Scale Bars: 10 μm. (D) Percentage of starved wild-type and *ptr-18* mutant L1 larvae showing GRL-5::VENUS expression. Data were collected after 24 h L1 starvation and are presented as mean ± SD. Experiments were repeated three times, and n ≥50 animals were scored for each trial.
(PDF)

S7 Fig *mir-235* and *ptr-18* act in parallel pathways. (A) Loss of *mir-235* and *daf-16* does not affect the temporally-regulated internalization of GRL-7::mCherry::3xFLAG. GRL-7::mCherry::3xFLAG expression was assessed after 24 h L1 starvation. n ≥50 animals were scored. Experiments were repeated three times, and ≥50 animals were scored for each trial. Data are presented as mean ± SD. N.S.; Not statistically significant compared to wild type (Fisher’s exact test). (B) Loss of *mir-235* in *ptr-18* mutant animals enhances the quiescent defective phenotype. Data in Fig 1A are used for *ptr-18* mutants. The phenotype was scored after 5-day L1 starvation. Experiments were repeated ≥3 times, and ≥35 animals were scored for each experiment. Data are presented as mean ± SD. ****: p <0.0001 (Fisher’s exact test).
(PDF)
