# Supplementary figures and images for: Caenorhabditis elegans PTR/PTCHD PTR-18 promotes the clearance of extracellular hedgehog-related protein via endocytosis
Source: PLoS Genet. 2021 Apr 19;17(4):e1009457. doi: 10.1371/journal.pgen.1009457 (PMC8104386; doi:10.1371/journal.pgen.1009457)

S1 Fig  
Chiyoda et al.,

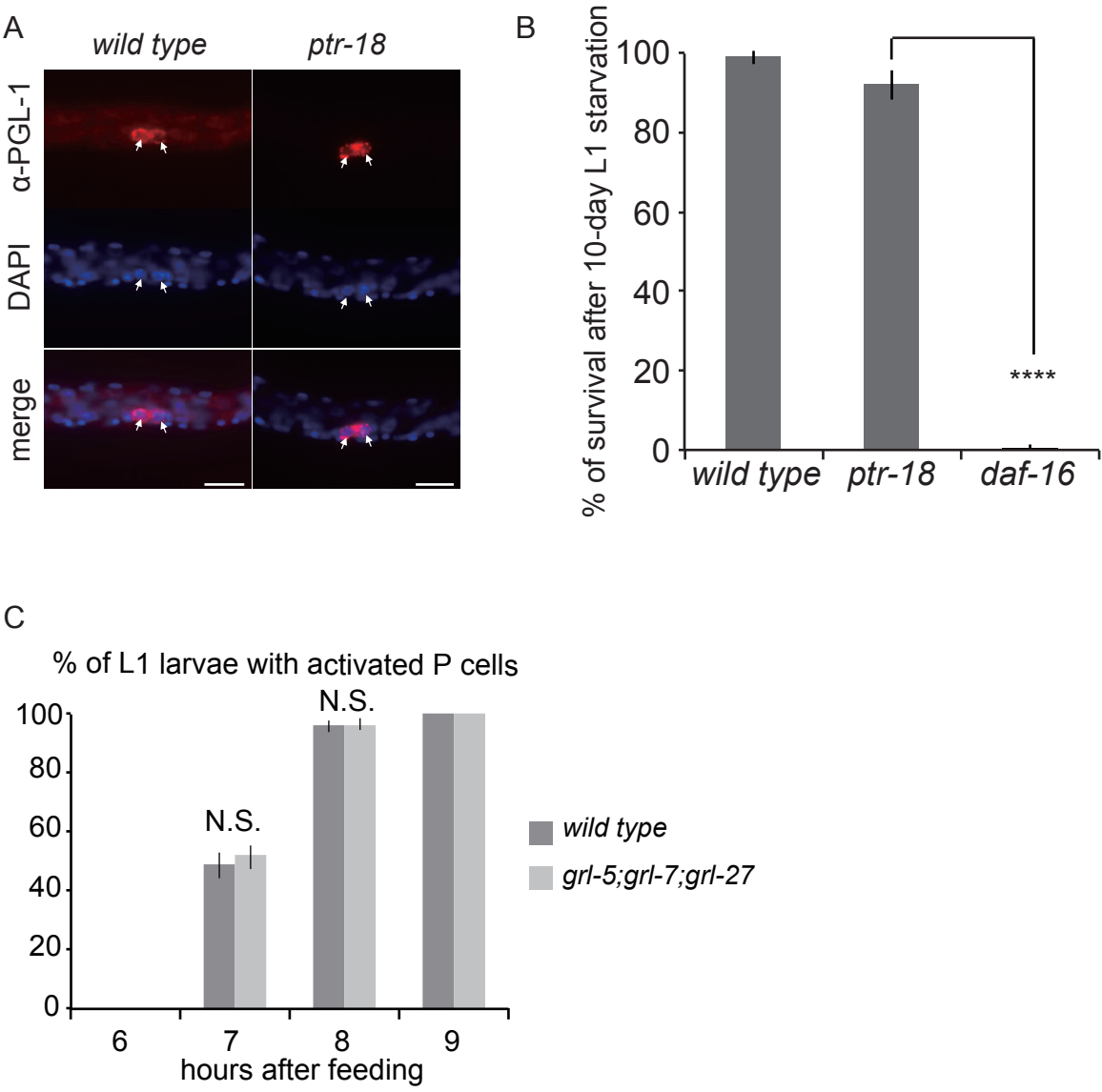

Supplement: S1 Fig — (A) Primordial germ cells, Z2 and Z3 (arrows), were visualized using anti-PGL-1 antibody. The absence of the proliferation of Z2 and Z3 was scored under the Nomarski microscopy after 5-day starvation in complete S media. Experiments were repeated three times, and n ≥35 animals were scored for each trial (see text). Scale bar: 10 μm. (B) Newly hatched L1 larvae were starved in cholesterol/EtOH-free complete S medium, and the viability was assessed after 10 days of culture. Experiments were repeated three times, and n ≥50 animals were scored for each trial. Data are presented as mean ± SD. ****: p <0.0001 (Fisher’s exact test). (C) Simultaneous loss of grl-5, grl-7, and grl-27 does not cause the delay of the timing of P cell activation. Data are presented as mean ± SD. Experiments were repeated three times, and n ≥50 animals were scored for each trial. N.S.: Not statistically significant (Fisher’s exact test). (PDF) [file pgen.1009457.s001.pdf]

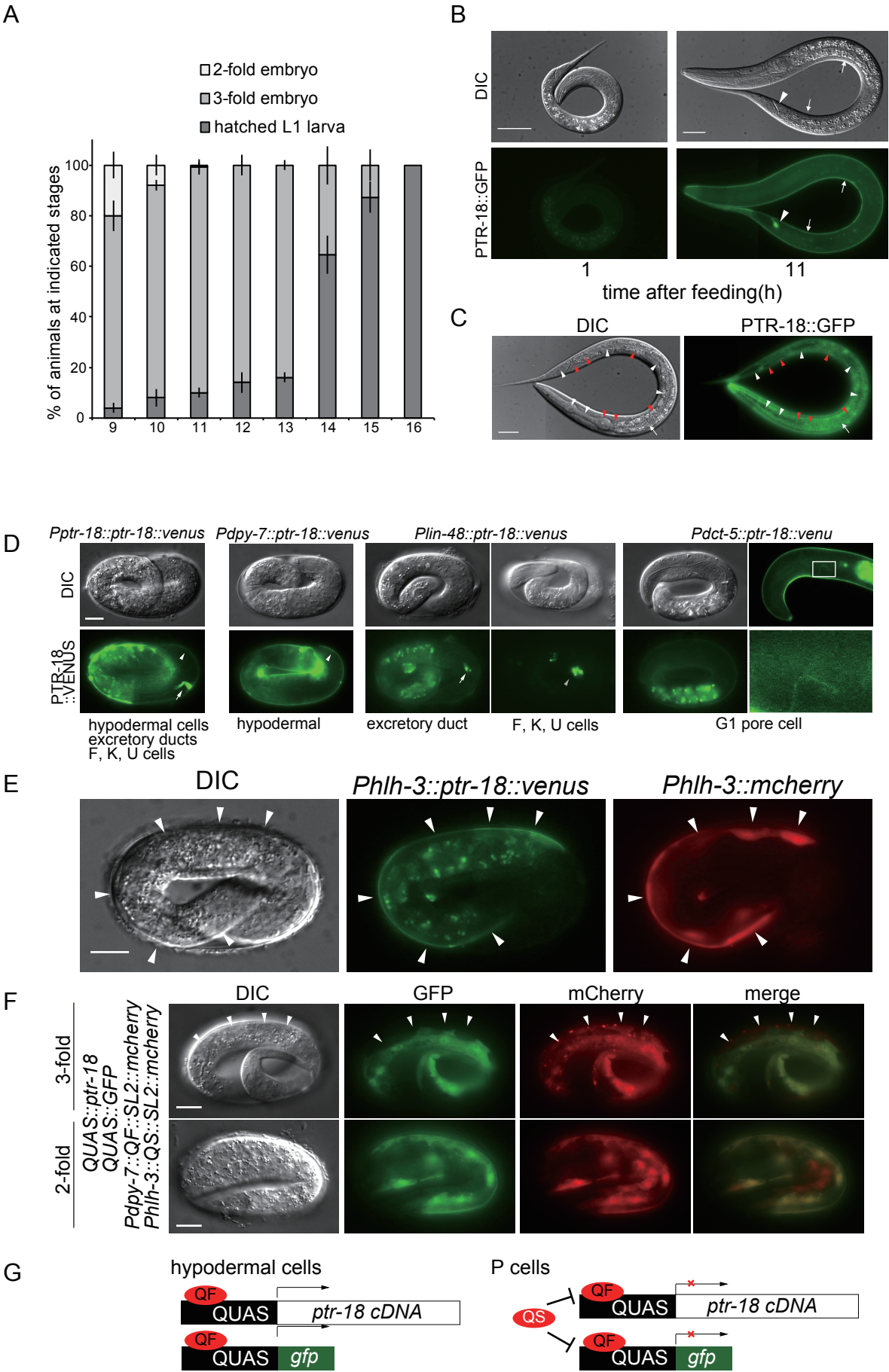

Supplement: S2 Fig — (A) Percentage of animals at each developmental stage after harvesting early embryos. Data are presented as mean ± SD. Experiments were repeated three times, and ≥50 animals were scored for each time point. (B) Hypodermal expression of PTR-18::GFP becomes detectable 11 h after the L1-arrested larvae are fed. Descendants of P cells (arrows) and F, K, and U cells (arrowhead) are indicated. Scale Bar: 10 μm. (C) Seam cells with or without PTR-18::GFP expression are shown in red and white arrowheads, respectively. The arrow indicates hypodermis. Scale Bar: 10 μm. (D) Expression of PTR-18::VENUS driven by the indicated promoters in 3-fold embryos. Expression of PTR-18::VENUS driven by the dct-5 promoter was only detectable after hatching. The arrow and arrowhead in the left panels indicate the excretory duct and hypodermal cells, respectively. Arrowheads in other panels point to cells indicated below. Types of cells expressing each reporter gene are indicated below each image. Scale Bar: 10 μm. (E) Expression of PTR-18::VENUS and mCherry driven by the hlh-3 promoter. P cells are indicated by arrowheads. Scale Bar: 10 μm. (F) PTR-18::VENUS is expressed in hypodermal but not P cells when driven by the Q system. The constructs introduced are indicated on the left. Note that the expression of PTR-18::VENUS was downregulated below detectable levels in P cells (arrowheads). Scale Bar: 10 μm. (G) Schematic of the hypodermis-specific expression of PTR-18::VENUS. The dpy-7 promoter drives the expression of transcriptional activator QF in both the hypodermal and P cells. However, QS expressed in P cells under the control of the hlh-3 promoter suppresses QF, resulting in the expression of PTR-18::VENUS only in the hypodermal cells. (PDF) [file pgen.1009457.s002.pdf]

S3 Fig  
Chiyoda et al.,

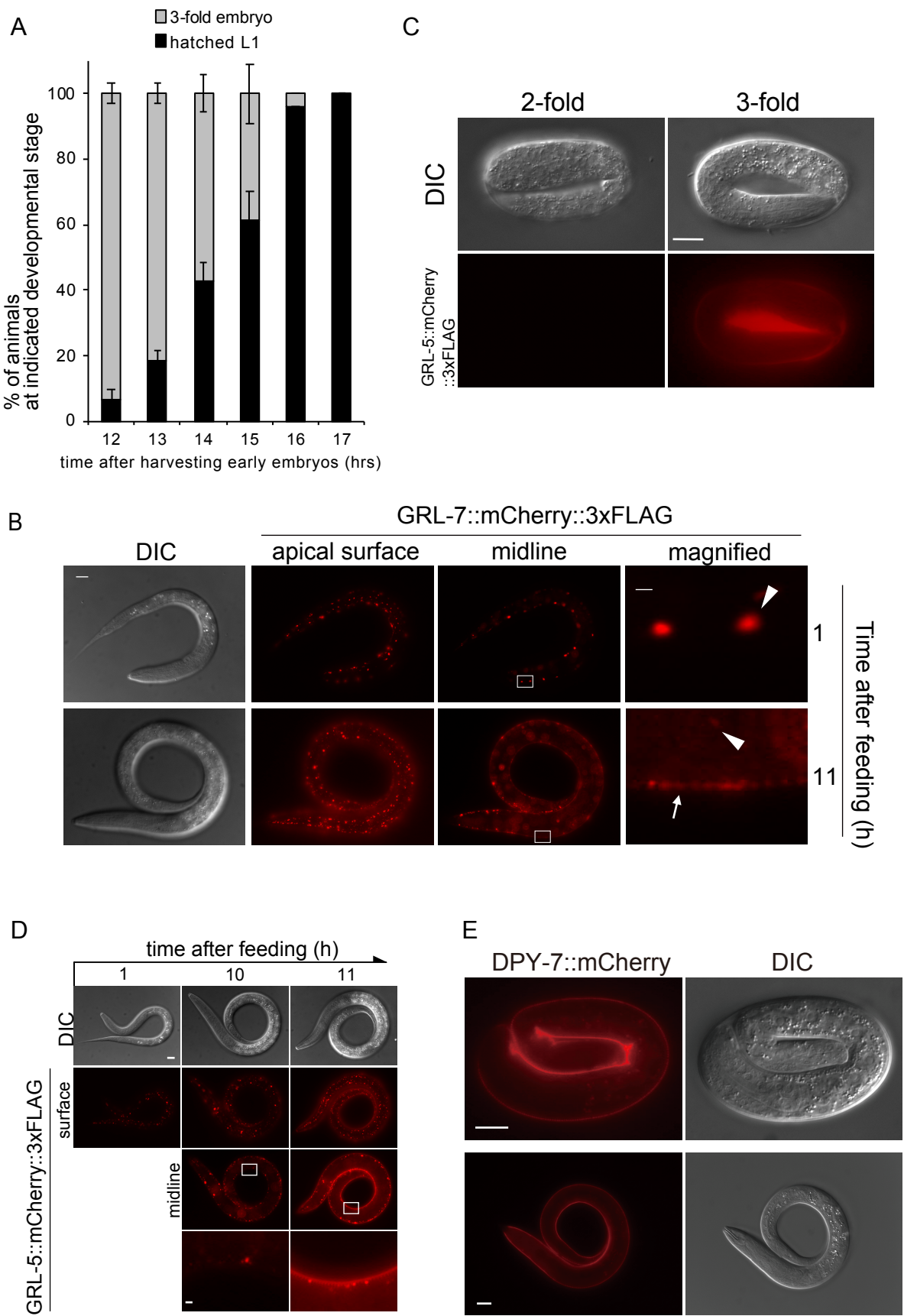

Supplement: S3 Fig — (A) Percentages of 3-fold embryos and L1 larvae after harvesting early embryos via the bleach treatment of gravid adults. Data are presented as mean ± SD. Experiments were repeated three times, and n ≥50 animals were scored for each trial. (B) Expression patterns of GRL-7::mCherry::3xFLAG in fed L1 larvae. Images shown are of L1 larvae 1 h (top panels) and 11 h (bottom panels) after the L1-arrested larvae were transferred to the fed condition. The photographs were taken with the focal planes on the apical surface (apical) and the ventral midline (midline). GRL-7::mCherry::3xFLAG localized at the vesicular structure and along the apical membrane are indicated by the arrowhead and arrow, respectively. The right panels are magnified views of the areas within the rectangle in the images on the left. Scale Bar: 10 μm (DIC image) and 2 μm (magnified image). (C) Expression of GRL-5::mCherry::3xFLAG during embryogenesis. Differential interference contrast and fluorescent images of embryos carrying the grl-5::mcherry::3xflag reporter gene. Scale Bar: 10 μm. (D) Expression of GRL-5::mCherry::3xFLAG in fed L1 larvae. Photographs show fed L1 larvae at indicated hours after the L1-arrested larvae were fed. Note that GRL-5::mCherry::3xFLAG accumulates at the apical surface of the whole body 11 h after the transfer. Photographs at the bottom are magnified views of the part of the above ones (indicated by the rectangles). All photographs showing GRL-5::mCherry::3xFLAG expression were taken at the same exposure time for comparison. Scale Bar: 10 μm (DIC image) and 1 μm (magnified image). (E) Expression of DPY-7::mCherry in 3-fold embryos (top panels) and newly hatched L1 larvae (bottom panels). The photograph of the L1 larva was taken after 24 h L1 starvation. Scale Bar: 10 μm. (PDF) [file pgen.1009457.s003.pdf]

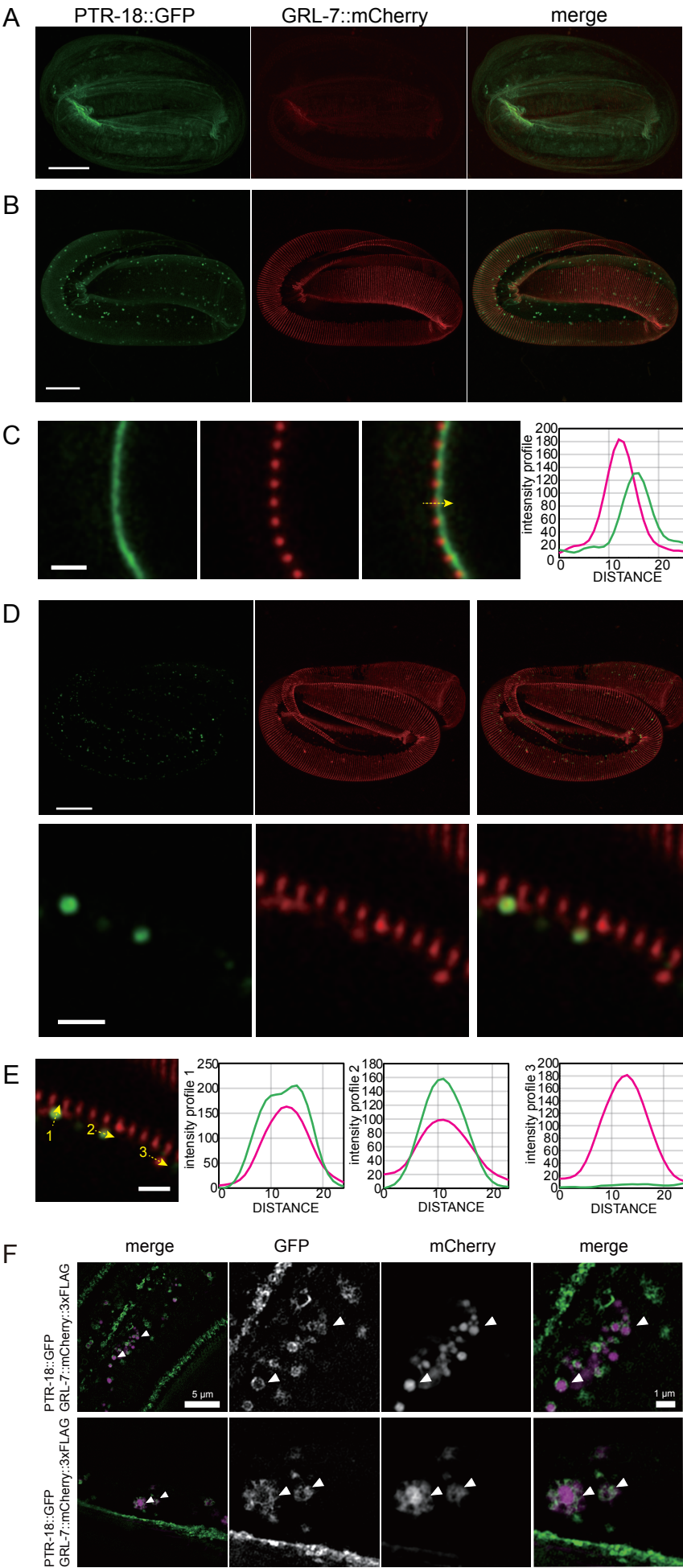

Supplement: S4 Fig — Super-resolution images showing the expression of CRISPR-generated grl-7::mCherry reporter gene co-expressed with ptr-18::gfp derived from a fosmid. Trios of images below show PTR-18::GFP (green), GRL-7::mCherry (red), and both, respectively, from left to right. (A) Maximum intensity Z-projection image of an embryo showing apical distributions of PTR-18::GFP and GRL-7::mCherry. Scale bar: 10 μm. (B) Maximum intensity Z-projection image of an embryo showing apical and vesicular distributions of PTR-18::GFP and apical localization of GRL-7::mCherry. (C) Magnified view from a Z section of an embryo indicated in B. Intensity profiles are determined along the arrow in the merged image. Scale bar: 10 μm. (D) Maximum intensity Z-projection image of an embryo showing predominantly vesicular patterns of PTR-18::GFP and apical and vesicular distributions of GRL-7::mCherry. Scale bar: 10 μm (upper panel) and 1 μl (bottom panel). (E) Magnified view from a Z section of an embryo indicated in B. Intensity profiles are determined along the arrows in the merged image. Note that PTR-18::GFP only localized to GRL-7::Cherry-positive vesicles at the left and middle regions, but not at the right. Intensity profiles are determined along the corresponding arrows in the merged image. Scale bars: 1 μm. (F) PTR-18 and GRL-7 localize to the same compartment. Structured illumination microscopy of L4 worms expressing PTR-18::GFP and GRL-7::mCherry. PTR-18 and GRL-17 populate the same compartment. While PTR-18 is present on the limiting membrane, GRL-7 accumulates inside. The nature of the compartment likely changes during development, comparing the top and bottom panels. N = 8 animals. Arrowheads point to the PTR-18 and GRL-17 positive compartments. (PDF) [file pgen.1009457.s004.pdf]

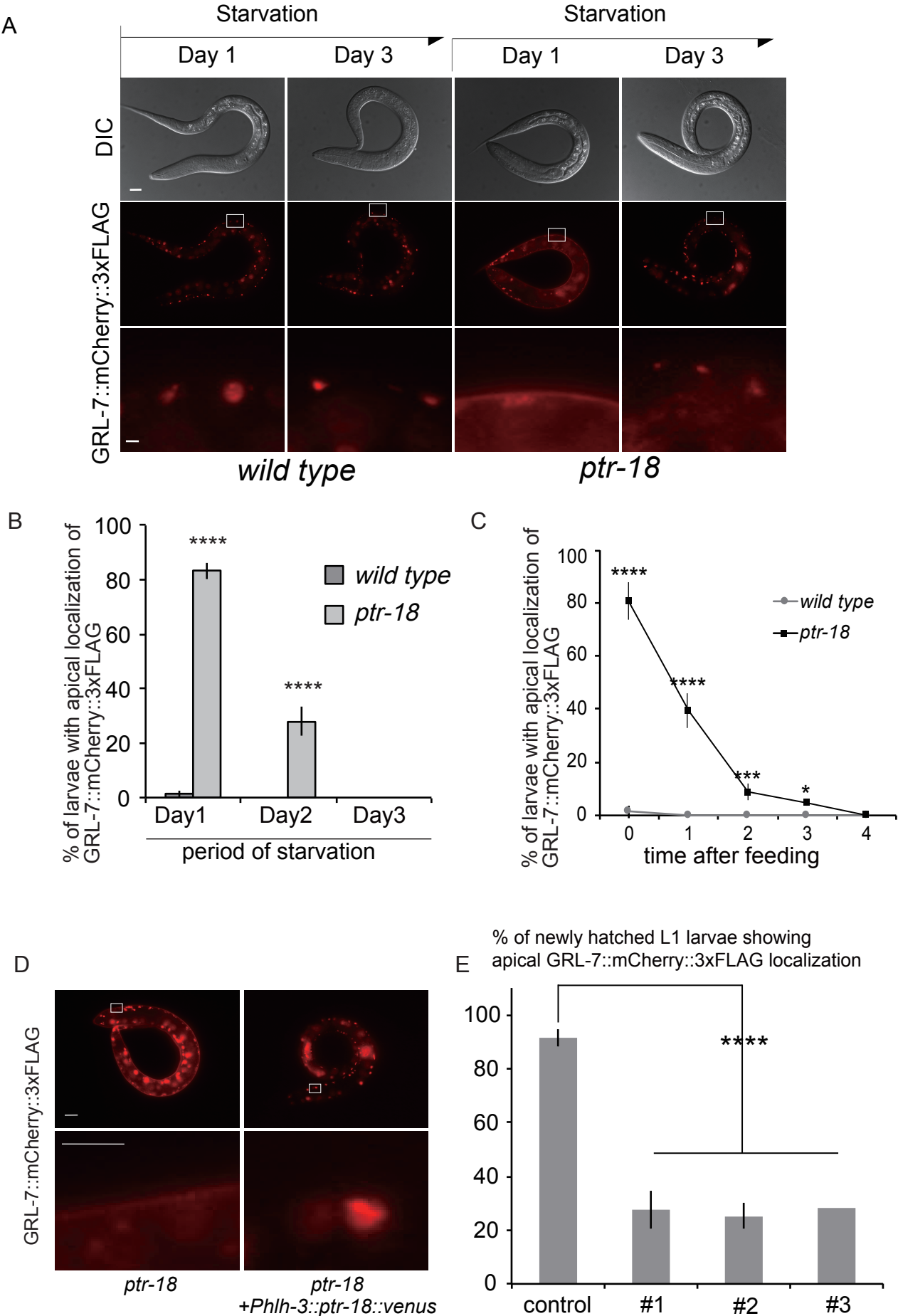

Supplement: S5 Fig — (A) GRL-7::mCherry::3xFLAG expression in starved wild-type and ptr-18 L1 larvae. Bottom panels are magnified views of the part of the middle panels (indicated by the rectangles). Scale bars indicate 10 μm (upper panel) and 1 μm (bottom panel). (B) Percentage of animals showing apical GRL-7::mCherry::3xFLAG localization during L1 starvation. GRL-7::mCherry::3xFLAG localization was scored at the indicated time points of L1 starvation in complete S medium. Note that the presence of cholesterol and ethanol in the medium does not affect the phenotype after 24 h L1 starvation (compare to Fig 7A and 7B, where L1 larvae were scored after 24 h L1 starvation in cholesterol-, ethanol-free complete S medium). Data are presented as mean ± SD. Experiments were repeated three times, and n ≥50 animals were scored for each trial. ****: p <0.0001 (Fisher’s exact test). (C) Percentage of animals showing apical GRL-7::mCherry::3xFLAG localization under the fed condition. GRL-7::mCherry::3xFLAG localization was scored at the indicated time points after feeding L1-arrested animals. Data are presented as mean ± SD. Experiments were repeated three times, and n ≥50 animals were scored for each trial. *: P<0.05, ***: P <0.001, and ****: p <0.0001 (Fisher’s exact test). (D) Expression of ptr-18::venus in P cells can suppress the prolonged apical localization of GRL-7::mCherry::3xFLAG in starved ptr-18(ok3532) L1 larvae. ptr-18 mutant larvae with or without the indicated transgenes after 24 h L1 starvation are shown. Scale Bars: 10 μm. (E) Percentage of newly hatched, ptr-18(ok3532) mutant larvae showing apical localization of GRL-7::mCherry::3xFLAG in the presence or absence of the transgenes that drives the expression of ptr-18::venus under the control of the P-cell specific hlh-3 promoter. Data are presented as mean ± SD. Experiments were conducted with three transgenic lines (#1 to #3) and repeated three times. n ≥50 animals were scored for each trial. ****: p <0.0001 (Fisher’s exact test) [file pgen.1009457.s005.pdf]

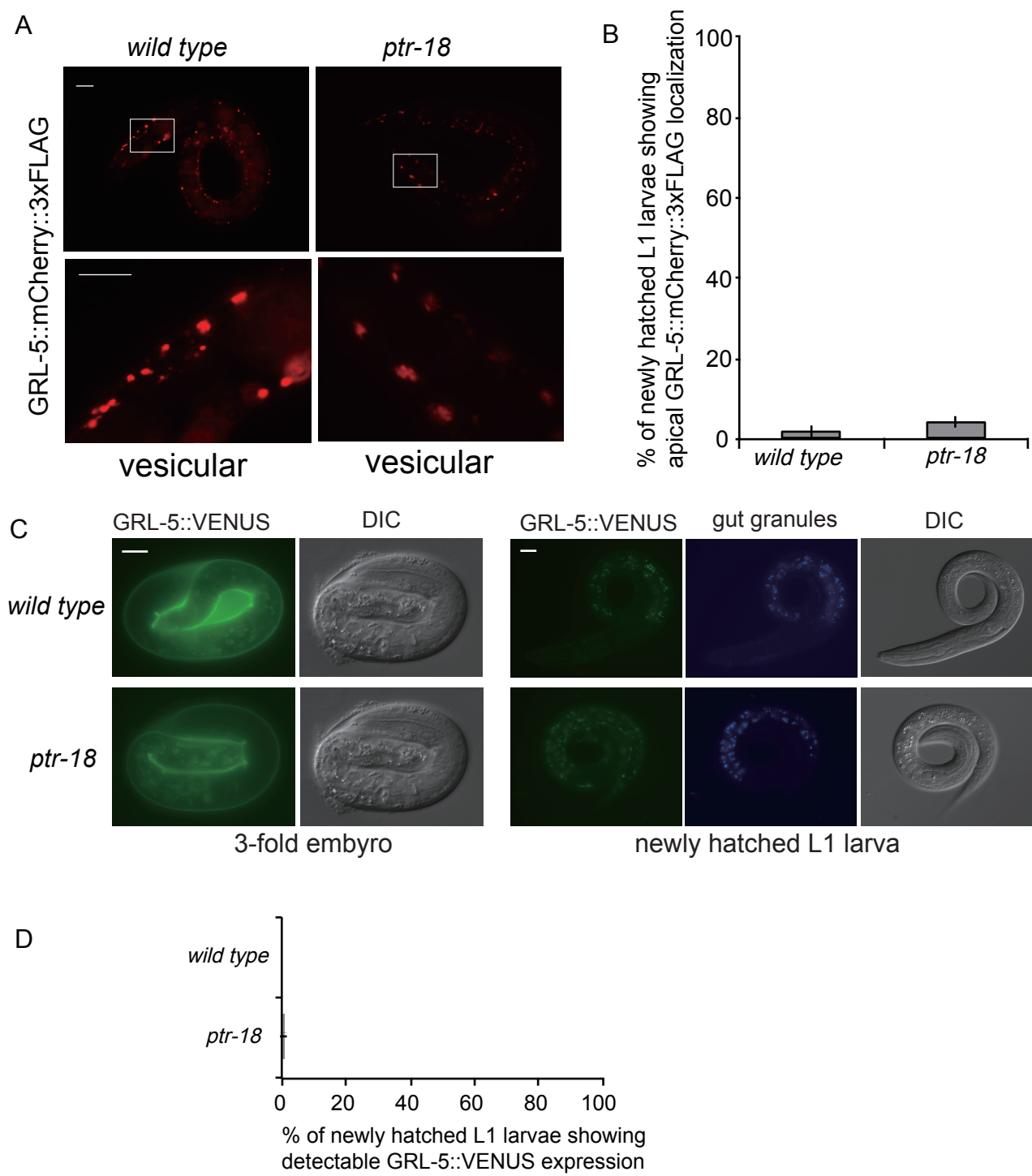

Supplement: S6 Fig — (A) Both newly hatched wild-type and ptr-18 mutant L1 larvae showing the vesicular distribution of GRL-5::mCherry::3xFLAG. The bottom panels are magnified views of the area within the rectangle from the images above. Scale bars indicate 10 μm (upper panel) and 1 μm (bottom panel). Photographs were taken with the same exposure time. B) Apical localization of GRL-5::mCherry::3xFLAG was rarely detected in the starved wild-type and ptr-18 mutant L1 larvae. n ≥50 animals were scored for each genotype. Data were collected after 24 h L1 starvation and are presented as mean ± SD. Experiments were repeated three times, and n ≥50 animals were scored for each trial. (C) Wild-type and ptr-18 mutant 3-fold embryos and L1 larvae after 24 h L1 starvation are shown. These animals carry transgenes that express GRL-5::VENUS fusion protein under the control of the native grl-5 promoter. Note that the fluorescence of L1 animals under the GFP filter is derived from gut granules. The presence of the transgene is marked by the co-injected plasmid that expresses mCherry in the hypodermis. Photographs were taken with the same exposure time. Scale Bars: 10 μm. (D) Percentage of starved wild-type and ptr-18 mutant L1 larvae showing GRL-5::VENUS expression. Data were collected after 24 h L1 starvation and are presented as mean ± SD. Experiments were repeated three times, and n ≥50 animals were scored for each trial. (PDF) [file pgen.1009457.s006.pdf]

S7 Fig  
Chiyoda et al.,

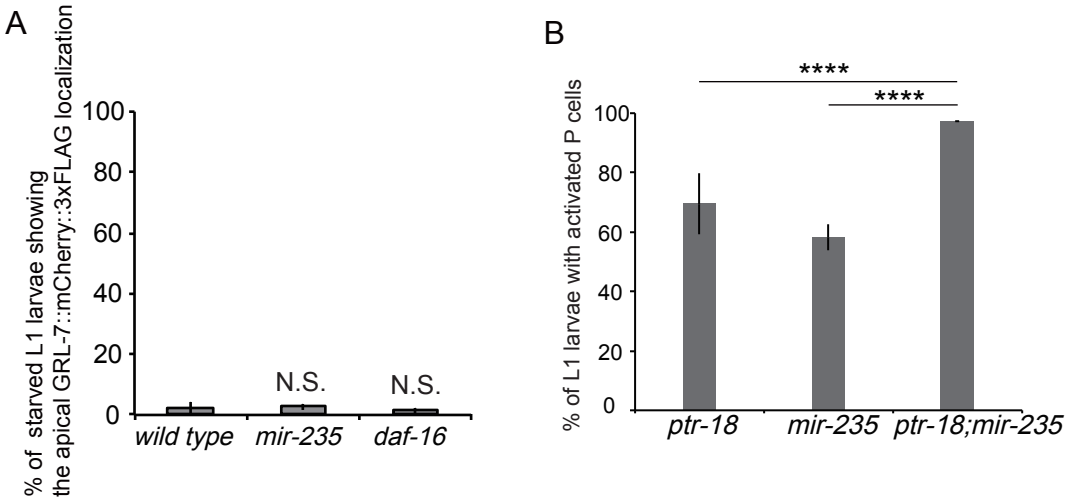

Supplement: S7 Fig — (A) Loss of mir-235 and daf-16 does not affect the temporally-regulated internalization of GRL-7::mCherry::3xFLAG. GRL-7::mCherry::3xFLAG expression was assessed after 24 h L1 starvation. n ≥50 animals were scored. Experiments were repeated three times, and ≥50 animals were scored for each trial. Data are presented as mean ± SD. N.S.; Not statistically significant compared to wild type (Fisher’s exact test). (B) Loss of mir-235 in ptr-18 mutant animals enhances the quiescent defective phenotype. Data in Fig 1A are used for ptr-18 mutants. The phenotype was scored after 5-day L1 starvation. Experiments were repeated ≥3 times, and ≥35 animals were scored for each experiment. Data are presented as mean ± SD. ****: p <0.0001 (Fisher’s exact test). (PDF) [file pgen.1009457.s007.pdf]
